# Obesity-driven mitochondrial dysfunction in human adipose tissue-derived mesenchymal stem/stromal cells involves epigenetic changes

**DOI:** 10.1038/s41419-024-06774-8

**Published:** 2024-06-01

**Authors:** Alfonso Eirin, Roman Thaler, Logan M. Glasstetter, Li Xing, Xiang-Yang Zhu, Andrew C. Osborne, Ronscardy Mondesir, Aditya V. Bhagwate, Amir Lerman, Andre J. van Wijnen, Lilach O. Lerman

**Affiliations:** 1 Division of Nephrology and Hypertension, Mayo Clinic, Rochester, MN USA; 2 Department of Cardiovascular Medicine, Mayo Clinic, Rochester, MN USA; 3 Department of Orthopedic Surgery, Mayo Clinic, Rochester, MN USA; 4 Department of Urology, The Affiliated Zhongda Hospital, Southeast University, Nanjing, China; 5 Department of Health Sciences Research, Mayo Clinic, Rochester, MN USA; 6 Department of Biochemistry, University of Vermont, Burlington, VT USA

**Keywords:** Mechanisms of disease, Stem-cell research

## Abstract

Obesity exacerbates tissue degeneration and compromises the integrity and reparative potential of mesenchymal stem/stromal cells (MSCs), but the underlying mechanisms have not been sufficiently elucidated. Mitochondria modulate the viability, plasticity, proliferative capacity, and differentiation potential of MSCs. We hypothesized that alterations in the 5-hydroxymethylcytosine (5hmC) profile of mitochondria-related genes may mediate obesity-driven dysfunction of human adipose-derived MSCs. MSCs were harvested from abdominal subcutaneous fat of obese and age/sex-matched non-obese subjects (*n* = 5 each). The 5hmC profile and expression of nuclear-encoded mitochondrial genes were examined by hydroxymethylated DNA immunoprecipitation sequencing (h MeDIP-seq) and mRNA-seq, respectively. MSC mitochondrial structure (electron microscopy) and function, metabolomics, proliferation, and neurogenic differentiation were evaluated in vitro, before and after epigenetic modulation. hMeDIP-seq identified 99 peaks of hyper-hydroxymethylation and 150 peaks of hypo-hydroxymethylation in nuclear-encoded mitochondrial genes from Obese- versus Non-obese-MSCs. Integrated hMeDIP-seq/mRNA-seq analysis identified a select group of overlapping (altered levels of both 5hmC and mRNA) nuclear-encoded mitochondrial genes involved in ATP production, redox activity, cell proliferation, migration, fatty acid metabolism, and neuronal development. Furthermore, Obese-MSCs exhibited decreased mitochondrial matrix density, membrane potential, and levels of fatty acid metabolites, increased superoxide production, and impaired neuronal differentiation, which improved with epigenetic modulation. Obesity elicits epigenetic changes in mitochondria-related genes in human adipose-derived MSCs, accompanied by structural and functional changes in their mitochondria and impaired fatty acid metabolism and neurogenic differentiation capacity. These observations may assist in developing novel therapies to preserve the potential of MSCs for tissue repair and regeneration in obese individuals.

## Introduction

Mesenchymal stem/stromal cells (MSCs) are adult stem cells residing in multiple tissues near blood vessels that constitute an endogenous cellular repair system endowed with anti-inflammatory and pro-angiogenic features [1]. These cells are the most frequently used cell type for regenerative medicine, and their autologous transplantation offers promise in several diseases [2]. However, the function of endogenous MSCs could be impaired in patients with morbidities, limiting their reparative capacity and autologous therapeutic potential.

Obesity is a global pandemic that imposes a large economic burden on healthcare systems [3] and is associated with increased cardiovascular morbidity and mortality [4]. Recent studies indicate that a central mechanism by which obesity raises cardiovascular risk is by accelerating tissue degeneration, while rendering endogenous repair systems and cell types, like MSCs, vulnerable to insults. We have demonstrated increased senescence and propensity for adipogenic and osteogenic differentiation in MSCs from pigs with diet-induced obesity [5]. Furthermore, obesity impairs the immunomodulatory capacity of human adipose tissue-derived MSCs both in vitro and in vivo [6]. However, the mechanisms by which obesity impairs human MSCs remain unknown.

Among their functions, MSCs can differentiate into neural precursors and/or mature neurons to promote neuroprotection and neurogenesis. Both experimental and clinical studies have shown that exogenous administration of MSCs from different sources confers neuronal protection in several diseases, including amyotrophic lateral sclerosis [7], autoimmune encephalomyelitis [8], Parkinson’s disease [9], and Alzheimer’s disease [10]. We postulated that obesity may compromise MSC neurogenic differentiation and that epigenetic mechanisms in MSCs might be pathologically modified to account for alterations in their biological properties.

The viability, plasticity, self-renewal, differentiation potential, and functionality of MSCs largely rely on the integrity and function of their mitochondria [11–13], organelles that not only produce cellular energy, but also modulate several important cellular functions including generation of reactive oxygen species (ROS), cell proliferation, survival, and apoptosis [14]. We have previously shown that obesity induces in swine MSCs mitochondrial dysfunction [15] associated with global genomic epigenetic alterations that primarily involve DNA hydroxymethylation [16]. This stable DNA modification results from hydroxylation of a methyl group attached to the C5 atom of cytosine (5mC) in cytosine-guanine dinucleotides to form transcriptionally activating hydroxymethylcytosine (5hmC) [17]. In this study, we tested the hypothesis that obesity alters DNA 5hmC levels in nuclear-encoded mitochondrial genes in human MSCs. We applied hydroxymethylated DNA immunoprecipitation and next-generation sequencing (hMeDIP-seq) to reveal that human obesity elicits site-specific DNA hydroxymethylation changes in nuclear-encoded mitochondrial genes in MSCs harvested from obese compared to age- and sex-matched non-obese subjects.

## Methods

### Study population

MSCs were harvested from abdominal subcutaneous fat collected from obese and age/sex-matched non-obese subjects (*n* = 5 each) during bariatric or kidney donation surgeries, respectively [18]. Informed written consent was obtained after approval of the Institutional Review Board of the Mayo Clinic. Entry criteria for obese patients included age 18–80 years and body mass index (BMI) > 30 kg/m² without or with obesity-related co-morbidities. Entry criteria for non-obese controls included age >18 years, BMI < 30 kg/m², and healthy overall state. Exclusion criteria for both groups included pregnancy, chronic inflammatory disease, active malignancy, recent stroke or myocardial infarction, solid organ transplant recipients, immunosuppressive treatment, or blood thinners/chronic anticoagulant therapy. Blood and urine samples were collected and total cholesterol, triglycerides, high-density lipoprotein (HDL), low-density lipoprotein (LDL), fasting glucose, hemoglobin-A1C (HbA1c), aspartate aminotransferase (AST), uric acid, serum creatinine, and urine protein levels were assessed by standard procedures. Estimated glomerular filtration rate (eGFR) was calculated using the chronic kidney disease epidemiology collaboration formula [19]. Blood pressure (systolic, diastolic, mean) and use of concomitant medication were also recorded.

### MSC harvesting and characterization

Abdominal fat samples (0.5–2.0 g) were digested in collagenase-H and filtered. MSCs were then cultured for 3 weeks, as previously described [20–22] to reach passage-3 following the protocol of our clinical studies [23, 24]. All MSCs were cultured in the same Advanced Minimum Essential Medium supplemented with 5% platelet lysate. Cells were characterized using flow cytometry (Amnis FlowSight Millipore) for expression of common MSC markers (CD73+, CD90+, CD105+, CD34−, CD45−), and trilineage differentiation was verified [25]. These cells were subsequently used for hMeDIP-seq, RNA-seq, and in vitro studies (e.g., proliferation, migration, neurogenic differentiation, etc.).

### hMeDIP-seq

DNA was extracted from MSCs using the DNeasy Blood & Tissue Kits (Qiagen, Cat#: 69504) with RNase treatment following the manufacturer’s instructions [18, 26, 27], quantitated by Nano-drop instrument, and diluted into 100 ng/μl with TE buffer. The aliquot (100 μl) of diluted gDNA was sonicated using the Bioruptor® Pico (Diagenode, Seraing, Belgium) for 7–10 cycles of 30 s on and 30 s off. The size of fragmented DNA was analyzed by the Fragment analyzer (Advanced Analytical Technologies, Ankeny, IA) using the High Sensitivity NGS Fragment Analysis Kit (Cat#: DNF-486). Fragmented DNA with an average size of 200 bp was denatured at 95 °C for 10 min. 2.5–5 µg of DNA in 1X DIP buffer (10 mM sodium phosphate, pH7.0, 140 mM NaCl, 0.05% Triton X-100) was incubated with 1 µg of anti-5hmC antibody generated from the hybridoma clone EDL HMC 1A (equivalent to the antibody (Millipore, Cat#: MABE1093)) for 3 h at 4 °C. Protein-G Dynabeads (ThermoFisher, Cat#: 10003D) were added, and the reactions were further incubated at 4 °C on a rotator overnight. Beads-antibody-DNA complexes were extensively washed by DIP buffer and TE buffer. Enriched DNA fragments were eluted from the beads, purified with the ssDNA/RNA Clean & Concentrator Kit (Zymo Research, Cat#: D7010) and quantified using the Qubit ssDNA High Sensitivity assay (Thermo Scientific, Cat#: Q10212). Libraries were prepared from input and enriched DIP DNAs by the ACCEL-NGS® 1S Plus DNA Library kit (Swift Bioscience, Cat#: 10024) [28] and sequenced to 51 base pairs from both ends on an Illumina HiSeq4000 instrument in the Mayo Clinic Medical Genomics Facility.

Bioinformatic analysis was performed by aligning paired-end sequenced FASTQ files to the human reference genome (hg38) using bowtie2 2.3.3.1 [29]. Duplicates were removed (PICARD 1.67, MarkDuplicates) and peaks identified using MACS2 [30]. Differential peak analysis was performed to determine sites of differential 5hmC coverage using the DiffBind 2.14.0 application package [31] and the HOMER 4.10 [32] peak annotation tool. The normalized data from DiffBind was generated using the bNormalized parameter of the DiffBind package. Additionally, HOMER assigns peaks to genes based on distances of peaks to the transcription start sites (TSS). Occasionally, however, peaks present within a gene may be assigned to an overlapping different nearest gene by HOMER. To correct for this, we used a consensus gene assignment approach between HOMER and BioMart to accurately identify transcripts and genes on which the peak was located. Specifically, if a peak was located within the body of a particular gene but was not assigned to that gene by HOMER, we identified the correct gene by intersecting the peak with the BioMart database. Gene symbols corresponding to 5hmC peaks were subsequently filtered for nuclear-encoded mitochondrial genes using MitoCarta2.0, an online inventory of 1,158 human genes encoding proteins with strong support of mitochondrial localization [33]. Heat maps of genes according to 5hmC levels in Obese-MSCs versus Non-obese-MSCs were generated using Morpheus (https://software.broadinstitute.org/morpheus/). Genomic distribution of hyper-hydroxymethylated [log_2_-fold-change (FC, Obese-MSCs/Non-Obese-MSCs)≥0.5, *p* ≤ 0.05] and hypo-hydroxymethylated [log_2_-FC (Obese-MSCs/Non-Obese-MSCs) ≤ −0.5, *p* ≤ 0.05] peaks was analyzed using Microsoft Excel based on their location (exon, intron, promoter, etc.) and distance to the transcription start site (TSS) and results adjusted for the potential confounding effect of triglyceride levels, antihypertensive drugs, and multivitamins.

### mRNA-seq, Western blot, and integrated (hMeDIP-seq/mRNA-seq) analysis

To explore whether obesity also elicited long-lasting effects on mitochondrial gene transcription, mRNA-seq analysis was performed [20, 34], filtered by MitoCarta2.0, and followed by an integrated (hMeDIP-seq/mRNA-seq) analysis. Differential expression analysis was performed using edgeR 3.20.1. Expression values for each gene were normalized by the total number of reads/sample (Counts per Million mapped reads, CPM). Mitochondrial genes were selected based on statistical significance (*p* ≤ 0.05) and biologically relevant FC (FC ≥ 1.4 is upregulated and FC ≤ 0.7 is downregulated).

To identify nuclear-encoded mitochondrial genes dysregulated at both the epigenetic (hMeDIP-seq) and expression (mRNA-seq) level, Venn diagrams were generated using Venny 2.1.0. Peaks of overlapping (upregulated with hyper-hydroxymethylated peaks and downregulated with hypo-hydroxymethylated peaks) mitochondria-related genes in Obese-MSCs versus Non-Obese-MSCs were visualized using Integrative Genomics Viewer (IGV) [35] and their function determined using the GeneCards® database (http://www.genecards.org/). Expression of proteins encoded by hypo-hydroxymethylated/downregulated genes, such as dodecenoyl-CoA Delta Isomerase (DCI, ThermoFisher, cat#: PA5–79184, 1:1000), MECR (ThermoFisher, cat#: PA5–55967, 1:200), and coenzyme-A (CoA, ThermoFisher, cat#: PA5–117880, 1:500), as well as expression of proteins encoded by hyper-hydroxymethylated/upregulated genes, such as adenine nucleotide transporter (ANT)-1 (CellSignaling, cat#: 69569, 1:1000), SLC22A4 (LSBio, cat#: LS-C8100000-100, 1:1000), LAMC1 (CellSignaling, cat#: 92921, 1:1000), and COQ10B (ThermoFisher, cat#: BS-11656R, 1:1000) were determined by western blotting and adjusted by GAPDH (Abcam, cat#: ab8245, 1:5000.

### Mitochondrial structure and function

Mitochondrial structure was assessed using digital transmission electron microscopy (Phillips CM10) [36, 37] in Non-Obese- and Obese-MSCs. Because we found that genes associated with mitochondrial structural and functional damage were hyper-hydroxymethylated and upregulated in Obese-MSCs, mitochondrial structure was also assessed in Non-Obese- and Obese-MSCs treated with the epigenetic modulator Bobcat339 (C16H13Cl2N3O, 10 μM for 24 h, MedChemExpress, Cat#: HY-111558A), a selective cytosine-based Ten-11 translocation methylcytosine dioxygenase (TET) enzyme inhibitor that reduces DNA 5hmC abundance [38]. Cells were preserved in Trump’s fixative solution (4% formaldehyde and 0.1% glutaraldehyde in 0.1 M phosphate buffer), mounted on mesh grids, and stained with aqueous uranyl acetate and lead citrate. Representative MSCs (*n* = 20) were randomly selected, and mitochondrial area (nm²) and matrix density (1/mean gray values) were determined. Mitochondrial ROS production was measured by Mito-SOX (ThermoFisher, Cat#: M36008) [39], membrane potential determined by tetramethylrhodamine ethyl ester (TMRE, Cat#:T669) [40], and ATP production by colorimetric and fluorometric methods (Promega, cat#: G7570) [37] in Non-Obese- and Obese-MSCs untreated or treated with Bobcat339 or dimethyl alpha-ketoglutarate (DMαKG, Sigma-Aldrich Cat#: 349631, 5 mM), a co-factor that TET enzymes require to convert 5mC into 5hmC [41].

### MSC proliferation, migration, fatty acid metabolism, and neurogenic differentiation

MSC proliferation was assessed in Non-Obese- and Obese-MSCs using a Cell Imaging Multimode Reader (Cytation-5, BioTek Santa Clara, CA). MSCs were seeded in a 24-well plate (5 × 10^4^/well) and kept at 37 °C with 5% CO_2_. Cell confluence was captured hourly for 70 h, and data were analyzed using Gen5 software (Bio-Tek) [42].

MSC migratory function was tested using a QCMTM Colorimetric Cell Assay (EMD Millipore, Burlington, MA; Cat#: ECM508), according to the company’s standard protocol [43].

MSC neurogenic differentiation was assessed by β-III tubulin (eBioscience Cat#: 14-4510-82-AF488) and Nissl body staining (PromoCell). Because we found that genes involved in neurogenic differentiation were hypo-hydroxymethylated and downregulated in Obese-MSCs, Nissl body staining was repeated in Non-obese- and Obese-MSCs treated with DMαKG, whereas cells treated with Bobcat339 served as negative control. For neurogenic induction, MSCs were seeded into a fibronectin-coated plate using MSC Growth Medium-2 (Cat#: C-28009) and allowed to reach 60–80% confluency. Subsequently, cells were cultured with MSC Neurogenic Differentiation Medium (Cat#: C-28015) or control (MSC Growth Medium-2) and incubated for at least 3 days, followed by specific staining of neuronal Nissl bodies using Saccomanno Fixation Solution (Morphisto, Cat#13881.00250) and Nissl staining solution (0.5% cresyl violet).

Because genes related to fatty acid (FA) metabolism were downregulated and hypo-hydroxymethylated in Obese-MSCs, FA metabolism was also determined by liquid chromatography-tandem mass spectrometry (LC-MS/MS) metabolomic analysis of Non-obese- and Obese-MSCs treated or untreated with DMαKG or Bobcat339. Briefly, samples were treated with 1.5–2 mL cold methanol to the dish (methanol stored in a clean bottle at −20 °C), scrapped to dislodge them from the dish’s surface, and transferred to 2 mL tubes placed on dry ice. Samples were dried and reconstituted with aTRAQ Reagent 113-labeled Standard Mix, and FAs separated and detected by LC-MS/MS. The concentrations of eicosapentaenoic acid (EPA), docosahexaenoic acid (DHA), and linolenic, myristic, palmitoleic, arachidonic, linoleic, palmitic, oleic, elaidic, and stearic acids were established by comparing their ion intensity (121-labeled FAs) to their respective internal standards (113-labeled FAs) [44]. In addition, expression of the lipogenic factors Diacylglycerol O-Acyltransferase-1 (*DGAT1*), Fas Cell Surface Death Receptor (*FAS*), and Lipoprotein Lipase (*LPL*) and the lipolytic factors Patatin-Like Phospholipase Domain Containing-2 (*ATGL*), Lipase-E, Hormone Sensitive Type (*HSL*), and Monoglyceride Lipase (*MGL*) were measured by quantitative polymerase chain reaction (qPCR) using the ΔΔCt method, as described [45]. All primers are from ThermoFisher (DGAT1: Hs1020362, FAS: Hs00236330, LPL: Hs00173425, ATGL: Hs00982042, HSL: Hs00943410, and MGL: Hs00996004).

Finally, to assess the role of TET enzymes in mitochondria-related genes in human non-obese and obese-MSCs, we measured expression of *SLC25A4, SLC22A4, COQ10B, LAMC1, COASY, ECI1*, and *MECR* in Obese-MSCs versus Non-obese-MSCs untreated or treated with Bobcat339 or DMαKG. All primers are from ThermoFisher (Cat#: Hs00154037, Hs00268200, Hs00952238, Hs00267056, Hs00228787, Hs00157239, and Hs00211238, respectively).

### Statistical analysis

Statistical analysis was performed using JMP version 14 (SAS Institute, Cary, NC). Results were expressed as mean ± SD. The Shapiro-Wilk test was used to test for deviation from normality. Parametric (ANOVA/Student *t*-test) and nonparametric (Wilcoxon/Kruskal-Wallis) tests were used as appropriate followed by Student–Newman–Keuls test for multiple comparisons. Statistical significance was accepted for p ≤ 0.05.

## Results

### Systemic characterization of MSC donors

Age, sex, and blood pressure levels did not differ between the experimental cohort groups (Table 1). BMI and triglyceride levels were higher in obese individuals, whereas total-, HDL-, and LDL-cholesterol levels were similar to non-obese subjects. Fasting glucose, HbA1c, AST, uric acid, serum creatinine, eGFR, and proteinuria were also comparable between the groups, as were the number of antihypertensive, diuretic, statin/lipid-lowering, antibiotic, and multivitamin drugs. We note that the number of insulin/oral hypoglycemics and antidepressants/antianxiety drugs was higher in obese versus non-obese individuals, but it is uncertain whether these mitigants are relevant to MSC function [46, 47]. This systemic analysis of the patients indicates that the BMI value is the primary discriminator within our experimental cohort.

**Table 1:** Clinical, laboratory, and demographic data of non-obese and obese patients.

	Non-obese	Obese
Demographics		
Number	5	5
Age (years)	60.4 ± 7.2	56.6 ± 9.5
Sex (Male/Female)	2/3	2/3
Body Mass Index (kg/m²)	24.8 (24.3–26.8)	45.9 (39.4–48.3)*
Related laboratory measures:		
Systolic blood pressure (mmHg)	115.8 ± 20.2	118.0 ± 13.2
Diastolic blood pressure (mmHg)	65.8 ± 11.2	78.2 ± 5.7
Mean blood pressure (mmHg)	82.4 ± 12.6	91.5 ± 7.9
Total cholesterol (mg/dL)	198.3 ± 25.4	209.5 ± 7.8
Triglycerides (mg/dL)	73.6 ± 8.3	111.5 ± 10.6*
HDL (mg/dL)	55.3 ± 16.7	52.5 ± 2.1
LDL (mg/dL)	128.3 ± 8.7	125.5 ± 4.9
Fasting glucose (mg/dL)	92.0 (83.0–118.5)	96.0 (89.0–137.5)
HbA1c (mmol/mol)	4.9 ± 0.4	5.2 ± 0.3
AST (IU/L)	25.0 ± 2.6	22.3 ± 2.1
Uric acid (mg/dL)	5.0 ± 2.2	3.9 ± 2.2
Serum creatinine (mg/dl)	0.8 ± 1.3	0.9 ± 1.4
eGFR-MDRD (ml/min/1.73/m2)	75.8 ± 15.1	78.0 ± 11.0
Proteinuria (mg/24 h)	185.4 ± 83.2	259.5 ± 68.6
Concomitant medication (n/%):		
Antihypertensive drugs	1/10	2/20
Diuretic	0/0	0/0
Statins/lipid-lowering drugs	0/0	0/0
Insulin/oral hypoglycemics	0/0	3/30*
Antidepressants/antianxiety	0/0	3/30*
Antibiotics	0/0	1/10
Multivitamin	1/10	1/10

*HDL* high-density lipoprotein, *LDL* low-density lipoprotein, HbA1c A hemoglobin A1c, *AST*

aspartate aminotransferase, *eGFR* estimated glomerular filtration rate.

*p ≤ 0.05 vs. non-obese.

### Obesity alters the mitochondria-related hydroxymethylome profile of human MSCs

**Fig. 1:**
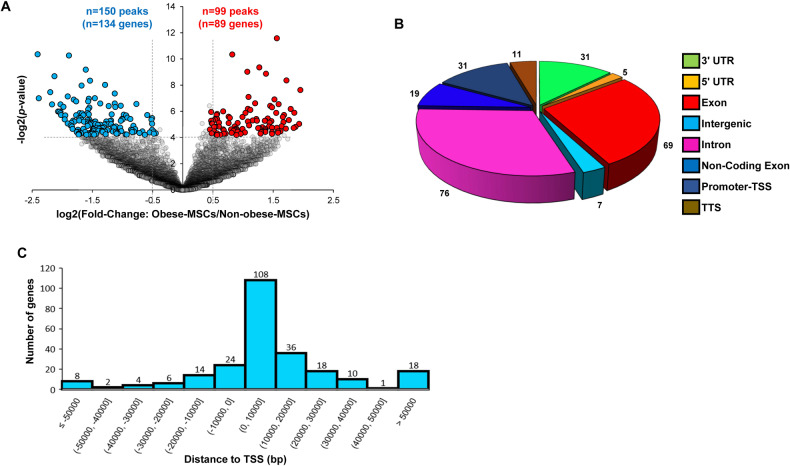
Obesity alters 5-hydroxymethylcytosine (5hmC) levels of mitochondria-associated genes in human adipose tissue MSCs. **A** Volcano plot of mitochondria-related genes with significant changes in 5hmC levels between Non-obese- and Obese-MSCs (*n* = 5 each). The y-axis corresponds to −log_2_ (*p* value), whereas the x-axis displays the log_2_ fold-change (Obese-MSCs/Non-obese-MSCs) value. Hyper-hydroxymethylated (*n* = 99; 89 genes) and hypo-hydroxymethylated (*n* = 150; 134 genes) peaks are indicated with red and blue dots, respectively. Cutoff values of *p* ≤ 0.05 and log_2_ fold-changes ≥0.5 or ≤−0.5 are indicated by gray dashed lines. **B** Genomic location annotations of hyper- and hypo-hydroxymethylated peaks. **C** Distribution across the gene body relative to the transcription start site (TSS).

To assess whether obesity has epigenetic effects on DNA hydroxymethylation of MSCs, we performed hMeDIP-seq. A total of 11,696 5hmC peaks in mitochondria-related genes were detected, among which 99 (corresponding to 89 genes) showed higher, and 150 (corresponding to 134 genes) showed lower, 5hmC levels in Obese-MSCs versus Non-obese-MSCs (Fig. 1A) and remained significant after adjustment for the potential confounding effect of triglyceride levels, antihypertensive drugs, and multivitamins (Table S1, Table S2). Hyper- and hypo-hydroxymethylated genes were primarily distributed in exonic, intronic, and promoter regions (Fig. 1B), and a large proportion of these genes were located relatively close to the TSS (Fig. 1C). These data show that obese and non-obese patients exhibit differences in the genomic distribution of 5hmC marks, reflecting obesity-related and DNA-based changes in epigenetic mechanisms.

### Epigenetic changes impact gene expression

**Fig. 2:**
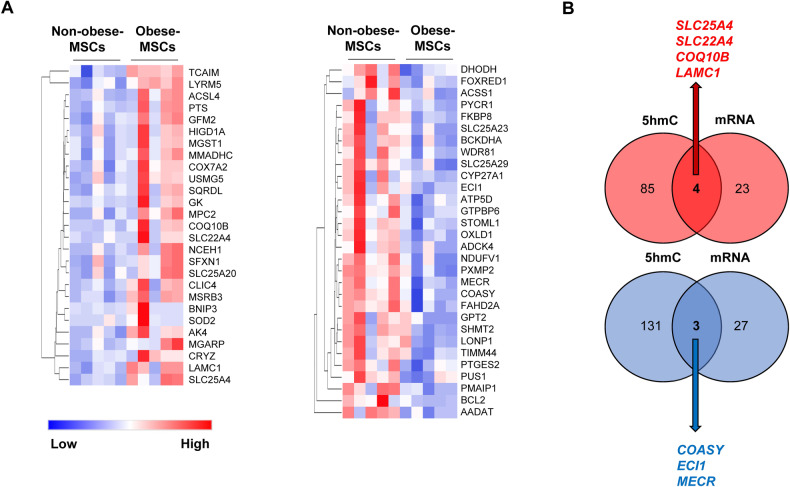
mRNA-seq and integrated hMeDIP-seq/mRNA-seq analysis. **A** Heat maps of mitochondria-associated genes upregulated (left) or downregulated (right) in Obese-MSCs compared to Non-obese-MSCs (*n* = 5 each). **B** Venn diagrams showing 4 genes (*SLC25A4*, SLC22A4, *COQ10B*, and *LAMC1*) with hyper-hydroxymethylated peaks that were upregulated (red) and 3 genes (*COASY*, *ECI1*, and *MECR*) with hypo-hydroxymethylated peaks that were downregulated (blue) in Obese-MSCs versus Non-obese-MSCs (*n* = 5 each).

**Fig. 3:**
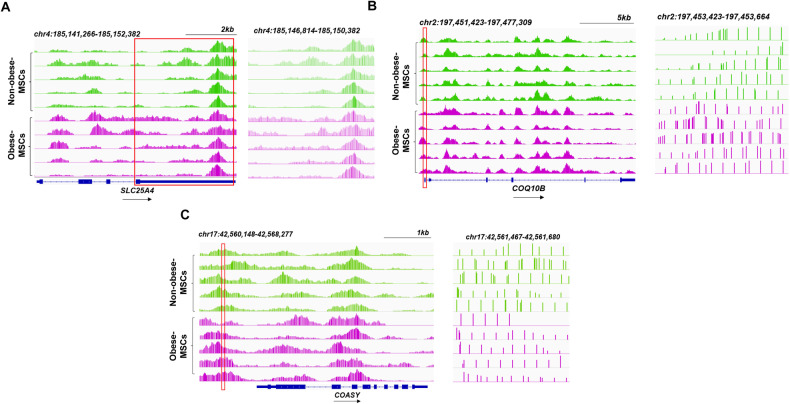
Visualization of 5hmC peaks. Representative integrative genomics viewer (IGV) tracks showing hyper-hydroxymethylated and hypo-hydroxymethylated peaks of *SLC25A4* (**A**), *COQ10B* (**B**), and *COASY* (**C**) in Obese-MSCs (purple) versus Non-obese-MSCs (green) (*n* = 5 each). All IGV tracks in a given comparison have the same scaling factor for the y-axis, and the scale of the x-axis is indicated in the upper right-hand region of each set of tracks. The region of the genome identified as differentially hyper- or hypo-hydroxymethylated is indicated by a red rectangle through the tracks. The RefSeq gene map is presented in blue at the bottom of each panel showing the overall gene structure.

To assess whether changes in 5hmC marks affect gene expression in MSCs, we correlated the hMeDIP-seq with mRNA-seq data. Transcriptome analysis identified 27 nuclear-encoded mitochondrial genes upregulated, and 30 downregulated, in Obese-MSCs versus Non-obese-MSCs (Fig. 2A). Integrated hMeDIP-seq/mRNA-seq analysis identified four genes [Solute Carrier Family 25 Member-4 (*SLC25A4*), SLC 22 Member-4 (*SLC22A4*), Coenzyme-Q10B (*COQ10B*), and Laminin Subunit Gamma-1 (*LAMC1*)] with hyper-hydroxymethylated peaks that were also upregulated, and 3 genes [Coenzyme-A Synthase (*COASY*), Enoyl-CoA Delta Isomerase-1 (*ECI1*), and Mitochondrial Trans-2-Enoyl-CoA Reductase (*MECR*)] with hypo-hydroxymethylated peaks that were also downregulated, in Obese-MSCs (Figs. 2B and 3). These genes were primarily implicated in ATP synthesis, redox activity, cell proliferation, migration, FA metabolism, and neurogenic differentiation (Table 2). Thus, obesity correlates with changes in the expression and activation of genes linked to metabolism and energy production, as well as growth and differentiation of MSCs. Protein expression of DCI (encoded by *ECI1*) followed the same direction of its parent gene, expression of MECR was higher in obese- versus non-obese-MSCs, but expression of coenzyme-A (CoA), encoded by *COASY*, was similar between the groups (Fig. S1). Similarly, the expression of adenine nucleotide transporter (ANT)-1 (encoded by *SLC25A4*) and SLC22A4 did not differ between the groups. However, in contrast to their gene expression, protein expression of COQ10B and LAMC1 was lower in obese- versus non-obese-MSCs.

**Table 2:** Mitochondria-related genes that were upregulated with hyper-hydroxymethylated peaks, or downregulated with hypo-hydroxymethylated peaks, in Obese-MSCs versus Non-obese-MSCs.

Gene symbol	Gene name	Cellular function
Upregulated/hyper-hydroxymethylated:		
SLC25A4	Solute Carrier Family 25 Member 4	ATP synthesis
SLC22A4	Solute Carrier Family 22 Member 4	ATP-dependent transport
COQ10B	Coenzyme Q10B	Proliferation/Redox activity
LAMC1	Laminin Subunit Gamma 1	Proliferation/migration
Downregulated/hypo-hydroxymethylated:		
COASY	Coenzyme A Synthase	Neurogenic differentiation
ECI1	Enoyl-CoA Delta Isomerase 1	Fatty acid metabolism
MECR	Mitochondrial Trans-2-Enoyl-CoA Reductase	Fatty acid metabolism

### Association with mitochondrial damage and MSC dysfunction

**Fig. 4:**
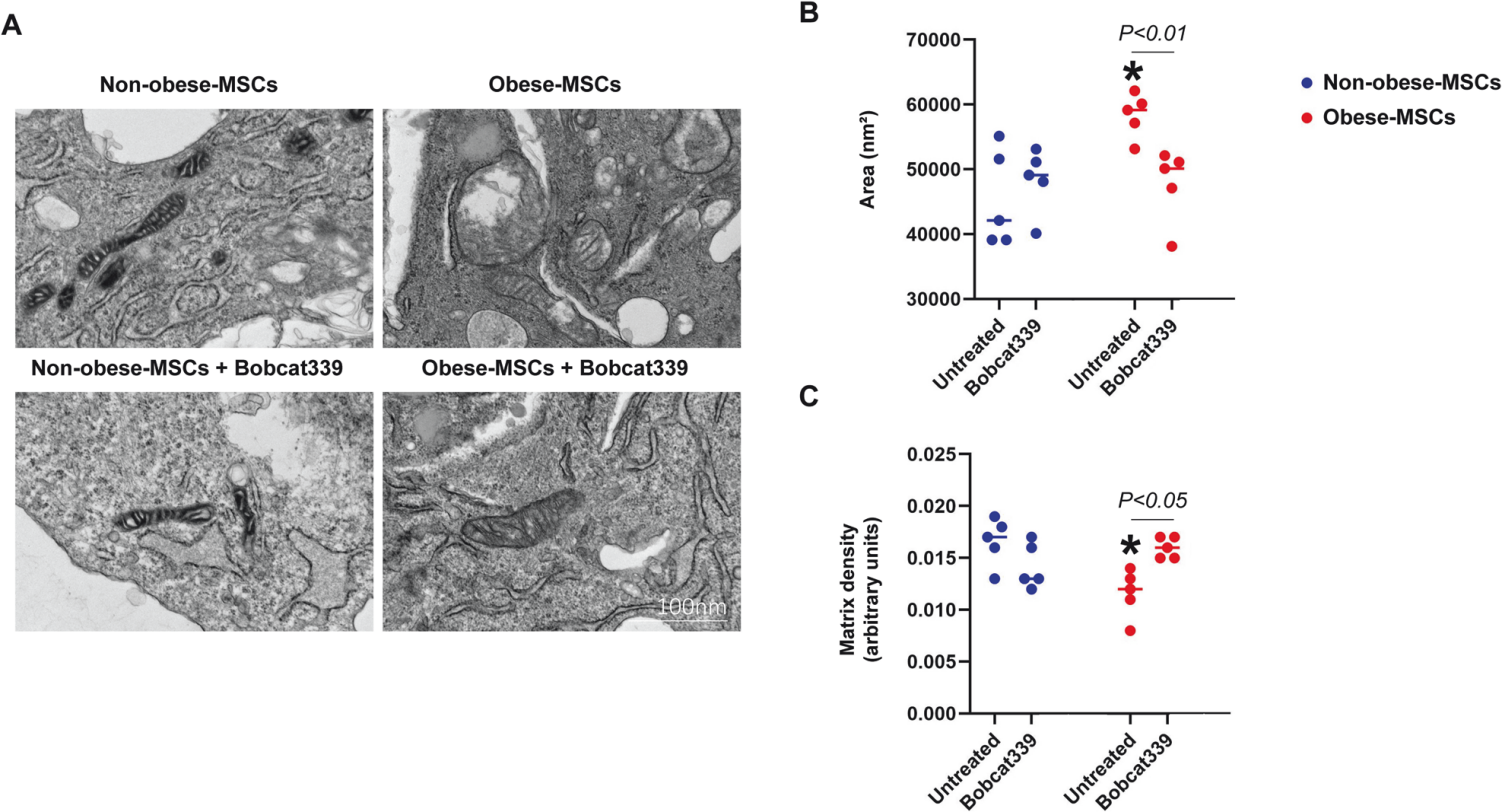
Obesity impairs mitochondrial structure in human MSCs. Representative transmission electron microscopy images (**A**) and quantification of mitochondrial area (**B**) and matrix density (**C**) in Non-obese- and Obese-MSCs untreated or treated with Bobcat339 (*n* = 5 each). *p value < 0.05 vs. Non-obese-MSCs (untreated).

**Fig. 5:**
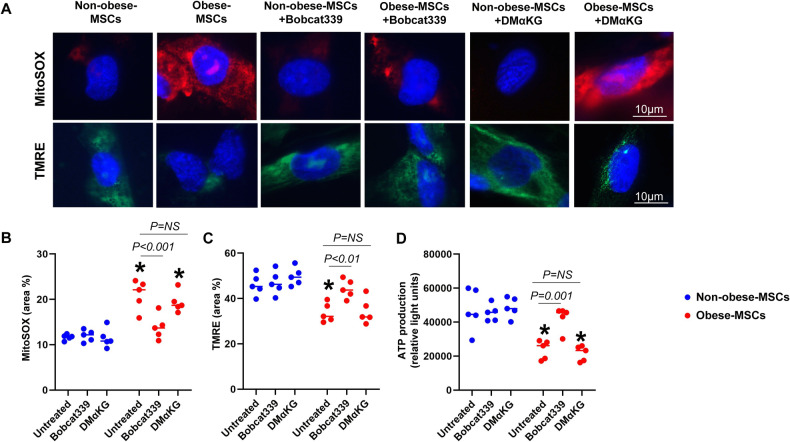
Obesity impairs mitochondrial function in human MSCs. Representative MitoSOX and tetramethylrhodamine ethyl ester (TMRE) staining (**A**) and quantification of mitochondrial reactive oxygen species (ROS) production (**B**), membrane potential (**C**), and ATP generation (**D**) in Non-obese- and Obese-MSCs untreated or treated with Bobcat339 or dimethyl alpha-ketoglutarate (DMαKG) (*n* = 5 each). *p value < 0.05 vs. non-obese-MSCs (untreated).

We then correlated the ultrastructural organization of mitochondria with the observed changes in mitochondrial gene expression. Under basal conditions, mitochondrial area was higher, and matrix density lower, in Obese-MSCs versus Non-obese-MSCs, reflecting a reduction in mitochondrial activity (Fig. 4). To assess the extent to which changes in DNA hydroxymethylation account for these changes in mitochondrial architecture, we co-incubated MSCs with an inhibitor (Bobcat339) that blocks the hydroxylase activity of TET enzymes, which improved mitochondrial morphology in Obese-MSCs (Fig. 4). In separate experiments, Obese-MSCs also exhibited higher production of mitochondrial ROS, but lower ATP levels and membrane potential, all of which improved with Bobcat339, but not by DMαKG (Fig. 5), establishing that changes in mitochondrial architecture are linked to reduced activity and to epigenetic modulation. These results underscore the potential of 5hmC-directed interventions to rescue mitochondrial structure and function in obese-MSCs.

**Fig. 6:**
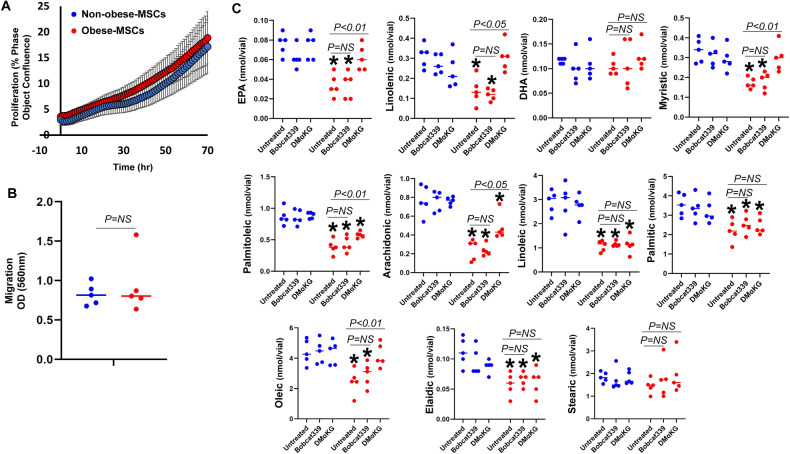
Obesity impairs fatty acid metabolism of human MSCs. Proliferation (**A** percent phase object confluence unit) and migration (**B** Colorimetric Cell Assay) of Non-obese and Obese-MSCs (*n* = 5 each). **C** Liquid chromatography-tandem mass spectrometry (LC-MS/MS) metabolomic analysis of the fatty acid (FA) metabolites eicosapentaenoic acid (EPA), docosahexaenoic acid (DHA), and linolenic, myristic, palmitoleic, arachidonic, linoleic, palmitic, oleic, elaidic, and stearic acids in non-obese- and Obese-MSCs untreated or treated with Bobcat339 or DMαKG (n = 5 each). *p value < 0.05 vs. Non-obese-MSCs (untreated). NS: non-significant (*p* > 0.05).

**Fig. 7:**
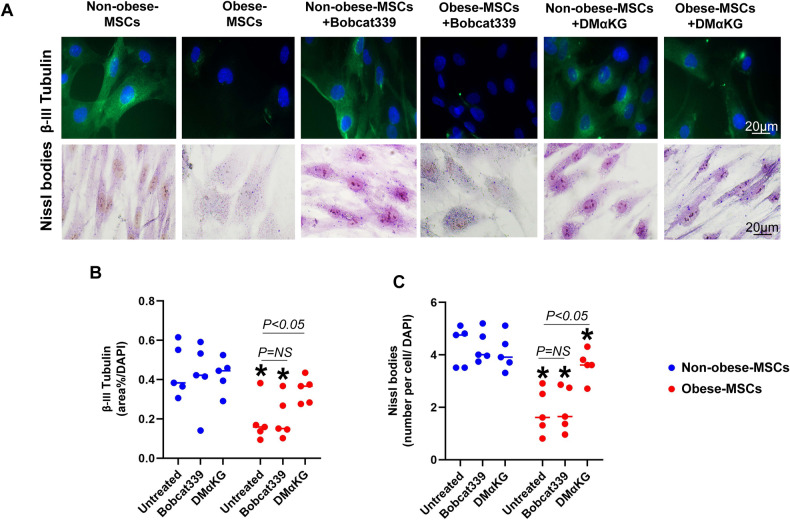
Obesity impairs neuronal differentiation of human MSCs. **A** Representative immunofluorescence β-III tubulin staining (green) and Nissl body staining (dark black-violet), and their quantification (**B-C**, respectively) in Non-obese- and Obese-MSCs untreated or treated with Bobcat339 or dimethyl alpha-ketoglutarate (DMαKG) (*n* = 5 each). *p-value < 0.05 vs. Non-obese-MSCs (untreated).

MSC proliferation and migration were similar between Non-obese-MSCs and Obese-MSCs (Fig. 6A, B), indicating that epigenetic differences and concomitant mitochondrial alterations do not affect mitotic cell division. Contrarily, obesity-related changes in the DNA hydroxymethylome decreased levels of the FA metabolites EPA, linolenic, myristic, palmitoleic, arachidonic, linoleic, palmitic, oleic, and elaidic acids (Fig. 6C), and decreased neurogenic differentiation in Obese-MSCs versus Non-obese-MSCs (Fig. 7A, B). These biological effects were mostly reversed in Obese-MSCs pre-treated with the TET cofactor DMαKG that is predicted to increase DNA hydroxymethylation but remained unchanged in cells treated with Bobcat339 (Fig. 7B). However, expression of lipogenic (DGAT1, FAS, LPL) and lipolytic (ATGL, HSL, MGL) factors did not differ between obese- and non-obese-MSCs (Fig. S2). Expression of *SLC25A4, SLC22A4, COQ10B*, and *LAMC1* was upregulated, while expression of *COASY, ECI1*, and *MECR* was downregulated in Obese- compared to Non-obese-MSCs (Fig. S11). Co-incubation of obese-MSCs with Bobcat339 decreased expression of *SLC22A4* and *LAMC1*, whereas co-incubation of obese-MSCs with DMαKG increased expression of *COASY, ECI1*, and *MECR*.

## Discussion

This study shows that obesity is associated with changes in both the hydroxymethylation and mRNA profiles of mitochondria-related genes of human adipose tissue-derived MSCs. Specifically, genes in which both gene activation and expression changed in the same direction were implicated in key cellular functions like ATP synthesis, redox activity, proliferation, migration, fatty acid metabolism, and neurogenic differentiation. Importantly, epigenetic and transcriptomic alterations in mitochondria-related genes were associated with mitochondrial structural and functional damage and with impaired FA metabolism and neurogenic differentiation, which were all restored in Obese-MSCs pre-incubated with pharmacological agents that target TET-dependent epigenetic modifications. These findings suggest that obesity-induced epigenetic changes may modify the phenotype and function of human MSCs.

Obesity impairs the function of MSCs [5] by increasing their senescence [48] and inflammatory signaling [49], decreasing pro-angiogenic activity [50], and altering cargo packed within MSC-derived extracellular vesicles [51]. Recently, we found that obesity imposes global 5hmC marks in swine MSCs, associated with MSC dysfunction [16]. The current study extends those observations to MSCs harvested from obese human subjects. We further took advantage of high-throughput hMeDIP-seq analysis, a tool with high resolving power, accuracy, and genome coverage, to identify 5hmC marks, which are generated by TET hydroxylases, across thousands of target regions [52].

We identified 99 peaks (89 genes) of hyper-hydroxymethylation and 150 peaks (134 genes) of hypo-hydroxymethylation in mitochondria-related genes from Obese- versus Non-obese-MSCs, which remained significant after adjustment for the potential confounding effect of triglyceride levels, antihypertensive drugs, and multivitamins, arguing against a major impact of these parameters in hydroxymethylation in human obese-MSCs. To determine if obesity-induced epigenetic changes are associated with altered expression of mitochondria-related genes, we performed an integrated hMeDIP-seq/mRNA-seq analysis and identified a select group of overlapping (higher or lower levels of both 5hmC and mRNA) mitochondria-related genes that are encoded by nuclear genomic DNA. We considered genes that were concurrently both activated and overexpressed, or vice versa, to be prominently implicated in differences between Obese- and Non-obese-MSCs.

For example, *SLC25A4* is a member of the mitochondrial carrier subfamily of solute carrier protein genes that encodes ANT-1, a mitochondrial inner membrane protein that transports ADP from the cytoplasm into the mitochondrial matrix and ATP in the opposite direction [53]. Therefore, obesity-induced activation and upregulation of this gene may alter ATP/ADP exchange across the mitochondrial inner membrane and compromise oxidative phosphorylation metabolism of MSCs.

Likewise, peaks of hyper-hydroxymethylation in *COQ10B* correlated with its higher expression levels in Obese-MSCs. The protein encoded by this gene (coenzyme CoQ10) functions as a membrane-localized antioxidant by protecting cells against lipid peroxidation [54]. However, CoQ10 might also increase intracellular ROS in healthy individuals, consistent with a pro-oxidant activity at cellular level [55], and increase the proliferative ability of human fibroblasts [56] and osteoblasts [57]. Thus, epigenetic upregulation of this gene might represent a defense mechanism but can also compromise both mitochondrial redox state and proliferative potential in Obese-MSCs, depending on their co-existing redox status.

Conversely, hypo-hydroxymethylated peaks in the FA metabolism genes *ECI1* and *MECR* and in the neuronal development gene *COASY* were associated with lower expression levels in Obese-MSCs. Pertinently, obesity poses a risk for neurodegenerative diseases, partly by inducing inflammation and mitochondrial injury [58]. Alas, nervous tissues have limited regeneration and recovery capabilities after injury [59]. MSCs are considered excellent endogenous therapeutic candidates for neurological diseases, due to their ability to differentiate into neural precursors and/or mature neurons [60]. Among them, adipose tissue-derived MSCs exhibit an electrophysiological response after neural induction, characteristic of mature-functional neurons [61, 62], associated with a significantly higher expression of neural markers and had a faster proliferation rate compared to other sources [59]. Mitochondria play a major role in FA metabolism and neuronal differentiation via regulation of the tricarboxylic acid (TCA) cycle [63]. Indeed, several mitochondria-protective interventions, such as antioxidants (MitoQ) [64], cardiolipin stabilizers (SS-31) [65], or mitochondrial fission inhibitors (Mdivi-1) [66], stimulate FA oxidation and reverse the progression of neurodegenerative disorders through the reinforcement of adult neurogenesis. *COASY* is an enzyme essential in CoA synthesis that is mainly present in the mitochondrial matrix and plays a critical role in neuronal development [67]. Consequently, epigenetic changes in the mitochondria-related gene *COASY* in MSCs imposed by obesity could alter their capacity to induce effective repair in individuals with neurodegenerative disorders.

Importantly, epigenetic and gene expression changes in *SLC25A4* and *COQ10B* may have a direct impact on mitochondrial structure and function. Obese-MSCs exhibited mitochondrial swelling (increased area), and their matrix density was considerably lower compared to Non-obese-MSCs, suggesting cristae remodeling and loss. This was associated with increased generation of ROS and decreased mitochondrial membrane potential, the main driver of ATP generation. Indeed, despite hyper-hydroxymethylation and consequently upregulation of genes implicated in ATP synthesis (e.g., *SLC25A4* and *SLC22A4*) ATP production decreased in Obese-MSCs, likely reflecting a compensatory mechanism to restore mitochondrial respiration. Congruently, the expression of corresponding proteins encoded by these genes was unaltered, suggesting opposing regulation to offset elevated gene activation.

Notably, epigenetic modulation with Bobcat339, which blunts DNA 5hmC, ameliorated mitochondrial structural damage and ROS production, and preserved membrane potential and ATP generation in Obese-MSCs, suggesting that TET enzymes could play an important role in modulating the expression of nuclear-encoded mitochondrial genes in human MSCs. Indeed, we found that co-incubation of obese-MSCs with Bobcat339 decreased expression of hyper-hydroxymethylated/upregulated genes, whereas co-incubation of obese-MSCs with DMαKG increased expression of hypo-hydroxymethylated/downregulated genes, underscoring the key role of TET enzymes in modulating mitochondrial gene hydroxymethylation in human MSCs. Yet, we cannot rule out the contribution of other epigenetics enzymes (e.g., EZH2, HAT1, LSD2, H3K4me3, H3K9me3, and H3K27me3), previously reported to be elevated in obese-MSCs [16], to alter mitochondrial gene expression and function imposed by obesity.

Interestingly, neither cell proliferation nor migration differed between the Non-obese and Obese groups, suggesting that the obesity-related epigenetics changes are relatively inconsequential for mitotic cell division of human MSCs. Neurogenic differentiation remained unaltered in Non-obese-MSCs but improved in Obese-MSCs treated with the 5hmC enhancer DMαKG. Furthermore, FA metabolism and neurogenic potential, which decreased in Obese-SCs, were mostly (albeit not invariably) restored to normal levels in Obese-MSCs pre-treated with DMαKG. This finding is consistent with the notion that epigenetic modulation of FA metabolism (e.g., *ECI1* and/or *MECR*) or neurogenic differentiation (e.g., *COASY*) genes may have partly contributed to the restoration of the functional capacity of Obese-MSCs. However, the expression of lipogenic and lipolytic factors was similar between Obese- and Non-obese-MSCs, arguing against a major role of obesity in modulating FA metabolism in human MSCs.

One potential limitation of our study is the relatively small sample size of our total cohort (n = 10), as often used in hMeDIP- and mRNA-seq studies due to the costs associated with these techniques [68–70]. This comparison by molecular analyses exceeds minimal standards for statistical analysis and permits firm biological conclusions but does not allow broadly generalizable conclusions. In addition, increased rate of duplicates and immunoprecipitation bias in hMeDIP-seq may result in data loss and potentially false positives [71]. Interestingly, many mitochondria-related genes in our integrated hMeDIP-seq/mRNA-seq analysis did not follow the same direction, and some of their protein products did not follow the same direction of their encoding genes. This may implicate alternative systems, such as other epigenetic changes (e.g., methylation and histone demethylation) as well as post-transcriptional (e.g., microRNAs, long-non-coding RNAs) and/or post-translational modifications (e.g., acetylation) in modulation of gene and protein expression and activation in human Obese-MSCs [72, 73]. These observations underscore the complexity and redundancy of regulatory mechanisms of cellular function. The precise mediator of epigenetic changes at chromosomal loci of nuclear-encoded mitochondria-associated genes in obese individuals remains to be determined but is likely linked to systemic metabolic states. The latter is conceptually linked to cardiovascular risk factors, such as hypertriglyceridemia [74] or hypertension [75], commonly associated with obesity, as well as medications (e.g., insulin/oral hypoglycemics and antidepressants/antianxiety drugs) that collectively may have partly accounted for changes in 5hmC levels in human Obese-MSCs. Furthermore, epigenetic changes observed in mitochondria-related genes of Obese-MSCs might have preceded or contributed to the development of obesity [76]. Thus, additional studies are needed to confirm our findings in a larger cohort to refine the role of epigenetic changes in Obese-MSC mitochondria-associated genes.

In summary, we observed that obesity is associated with changes in the 5hmC profile of mitochondria-related genes of human MSCs, accompanied by changes in the expression of mitochondria-related genes participating in ATP synthesis, redox activity, cell proliferation, migration, FA metabolism, and neurogenic differentiation. Importantly, these cells also showed impaired structure and function of MSC mitochondria and compromised fatty acid metabolism and neurogenic capacity. Therefore, these observations may reflect a key functional deficit in MSCs that may account for impaired tissue repair in obesity. Moreover, our study demonstrates the important role of epigenetic deregulation of mitochondrial genes in the nuclear genome in provoking mitochondrial dysfunction in human Obese-MSCs. Our findings may assist in developing novel strategies involving epigenetic modulators and/or mitoprotective drugs to preserve the therapeutic efficacy of MSCs in obese subjects. Further studies are needed to develop and test strategies to regulate the obesity-related epigenetic landscape of MSCs in vivo.

### Supplementary information

Supplemental Material

## Data Availability

The data that supports the findings of this study are available at: 10.6084/m9.figshare.23989263 and 10.6084/m9.figshare.23989266. Raw data (BAM files) were also deposited at Gene Expression Omnibus (GEO): GSE216948 and GSE216953.
