# Supplementary material for: Obesity-driven mitochondrial dysfunction in human adipose tissue-derived mesenchymal stem/stromal cells involves epigenetic changes
Source: Cell Death Dis. 2024 Jun 1;15(6):387. doi: 10.1038/s41419-024-06774-8 (PMC11144257; doi:10.1038/s41419-024-06774-8)
Supplement: Supplementary file 1 — Supplemental Material [file 41419_2024_6774_MOESM1_ESM.pdf]

SUPPLEMENTAL MATERIAL

Supplemental Figure Legends:

Figure S1. Expression of proteins encoded by hypo-hydroxymethylated/downregulated and hyper-hydroxymethylated/upregulated genes in Obese-MSCs. Protein expression of DCI (encoded by *ECI1*) followed the same direction of its parent gene, expression of MECR was higher in obese- versus non-obese-MSCs, but expression of coenzyme A (CoA), encoded by *COASY*, was similar between the groups. Similarly, expression of adenine nucleotide transporter (ANT)-1 (encoded by *SLC25A4*) and SLC22A4 did not differ between the groups. However, in contrast to their gene expression, protein expression of COQ10B and LAMC1 was lower in obese- versus non-obese-MSCs.

Figure S2. Obesity does not modulate the expression of genes linked to lipid metabolism in human MSCs. Expression (qPCR) of the lipogenic factors Diacylglycerol O-Acyltransferase 1 (*DGAT1*), Fas Cell Surface Death Receptor (*FAS*), and Lipoprotein Lipase (*LPL*) and the lipolytic factors Patatin Like Phospholipase Domain Containing 2 (*ATGL*), Lipase E, Hormone Sensitive Type (*HSL*), and Monoglyceride Lipase (*MGL*).

Figure S3. ANT-1 original uncropped western blot

Figure S4. SLC22A4 original uncropped western blot

Figure S5. COQ10B original uncropped western blot

Figure S6. LAMC1 original uncropped western blot

Figure S7. CoA original uncropped western blot

Figure S8. DCI original uncropped western blot

Figure S9. MECR original uncropped western blot

Figure S10. GAPDH original uncropped western blot

Figure S11. Expression of *SLC25A4, SLC22A4, COQ10B, LAMC1, COASY, ECI1,* and *MECR* in Non-obese- and Obese-MSCs untreated or treated with Bobcat339 or dimethyl alpha-ketoglutarate (DMαKG). *p-value < 0.05 vs. Non-obese-MSCs (untreated).

Fig. S1

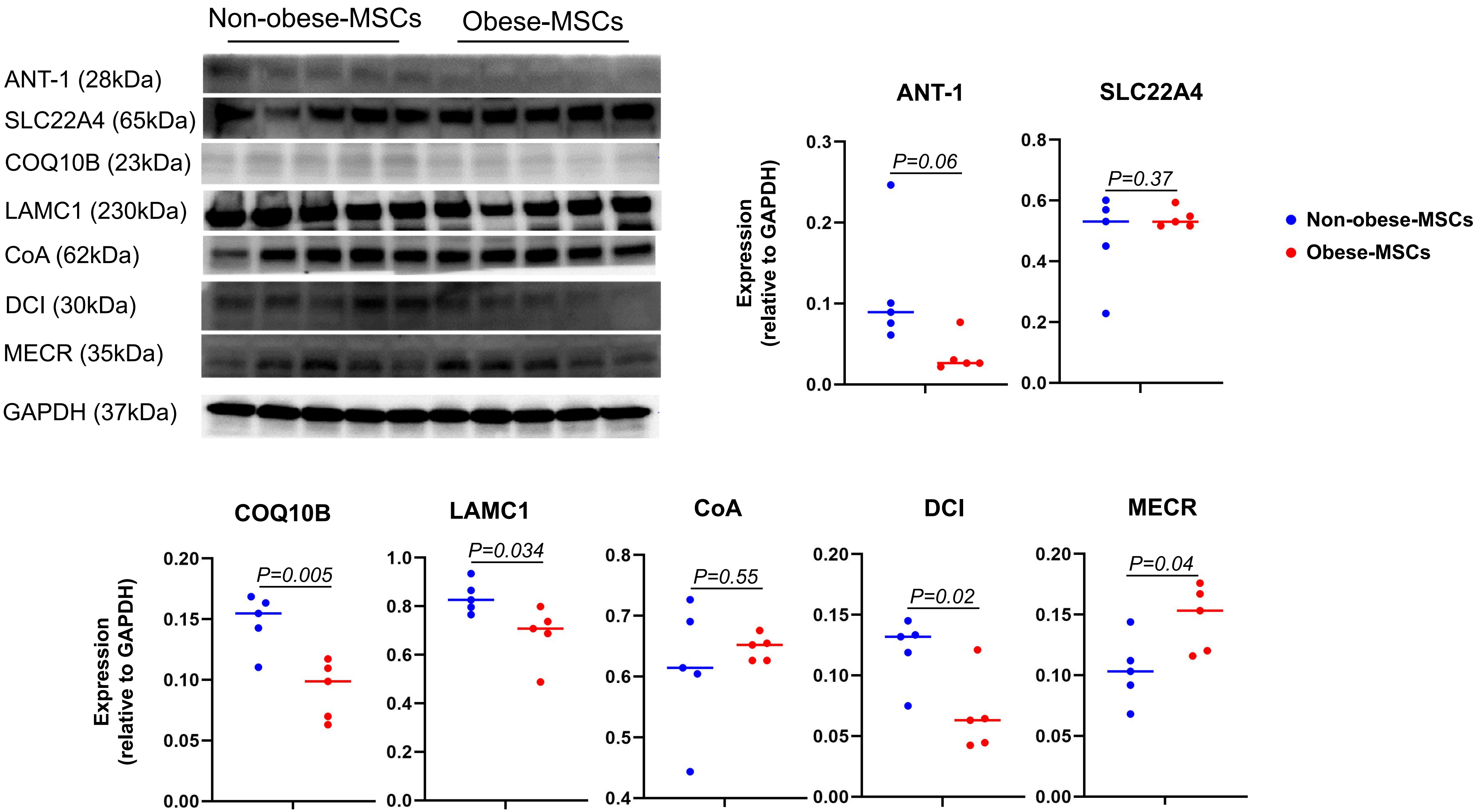

Non-obese-MSCs
Obese-MSCs
ANT-1 (28kDa)
SLC22A4 (65kDa)
COQ10B (23kDa)
LAMC1 (230kDa)
CoA (62kDa)
DCI (30kDa)
MECR (35kDa)
GAPDH (37kDa)
ANT-1
P=0.06
SLC22A4
P=0.37
Expression (relative to GAPDH)
Non-obese-MSCs
Obese-MSCs
COQ10B
P=0.005
LAMC1
P=0.034
CoA
P=0.55
DCI
P=0.02
MECR
P=0.04

Fig. S2

A

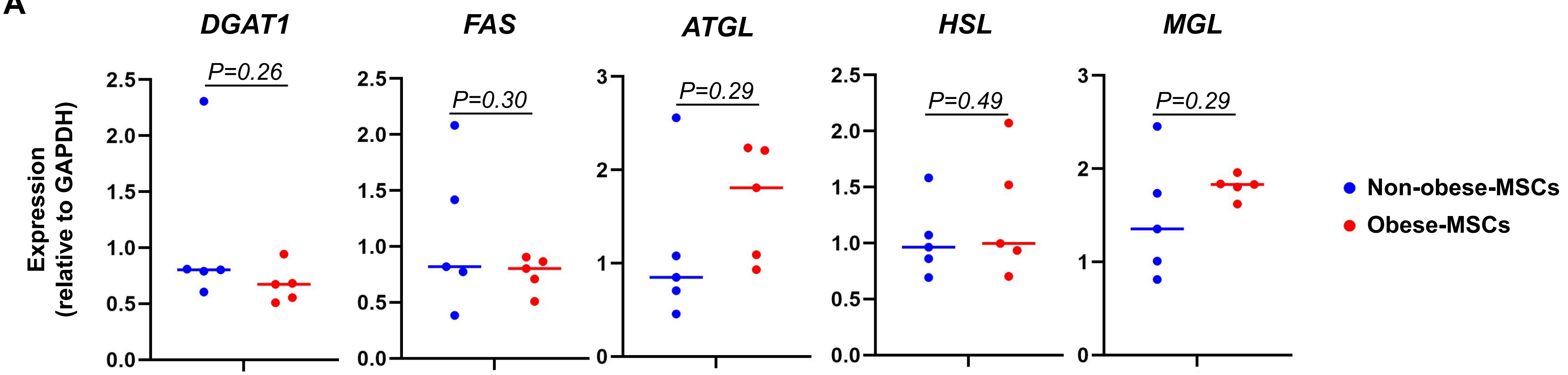
DGAT1
FAS
ATGL
HSL
MGL
Expression
(relative to GAPDH)
P=0.26
P=0.30
P=0.29
P=0.49
P=0.29
Non-obese-MSCs
Obese-MSCs

Fig. S3

ANT-1 (28kDa)

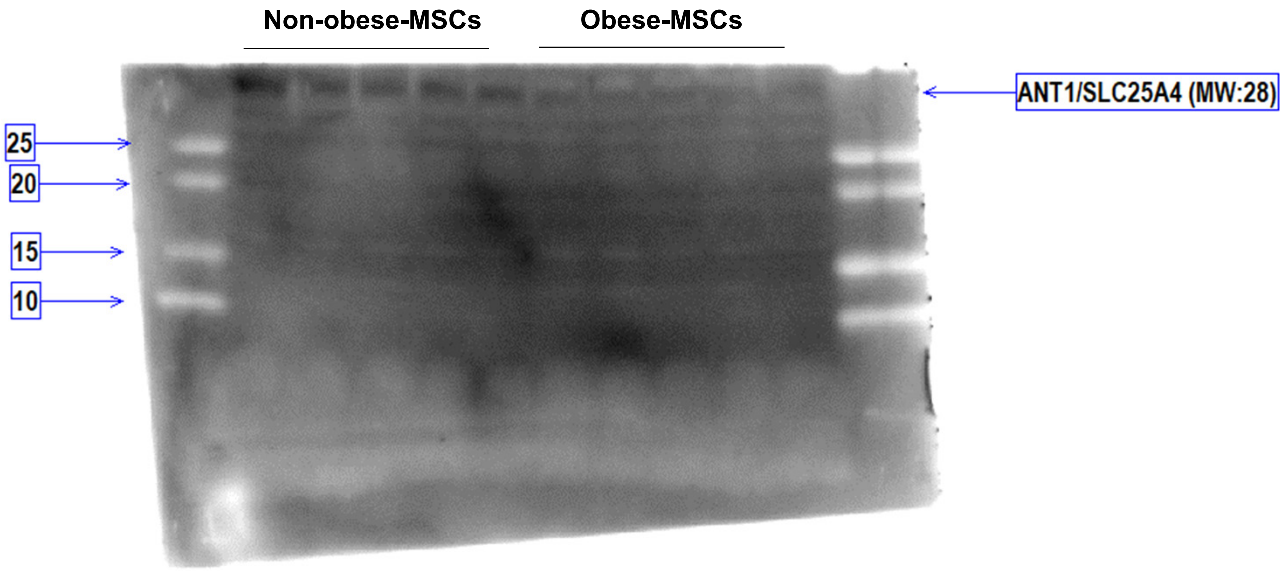

Fig. S4

SLC22A4 (65kDa)

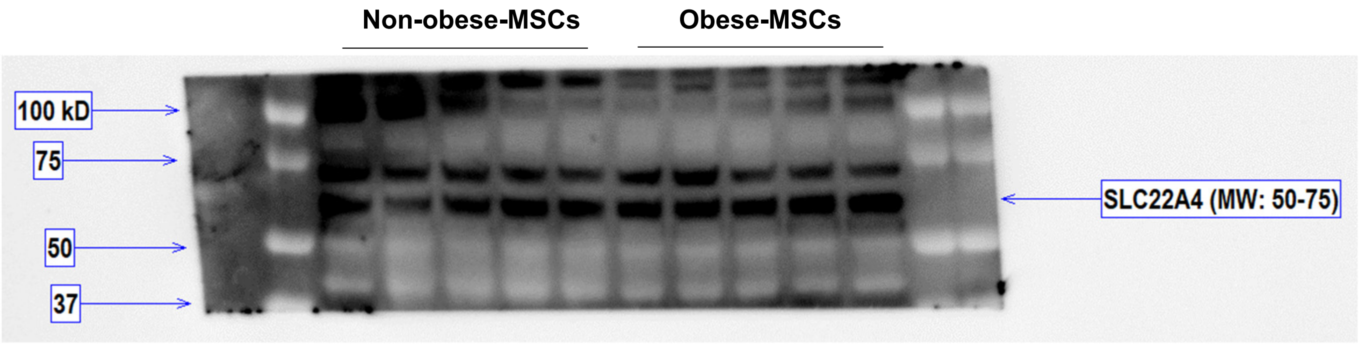

Fig. S5

COQ10B (23kDa)

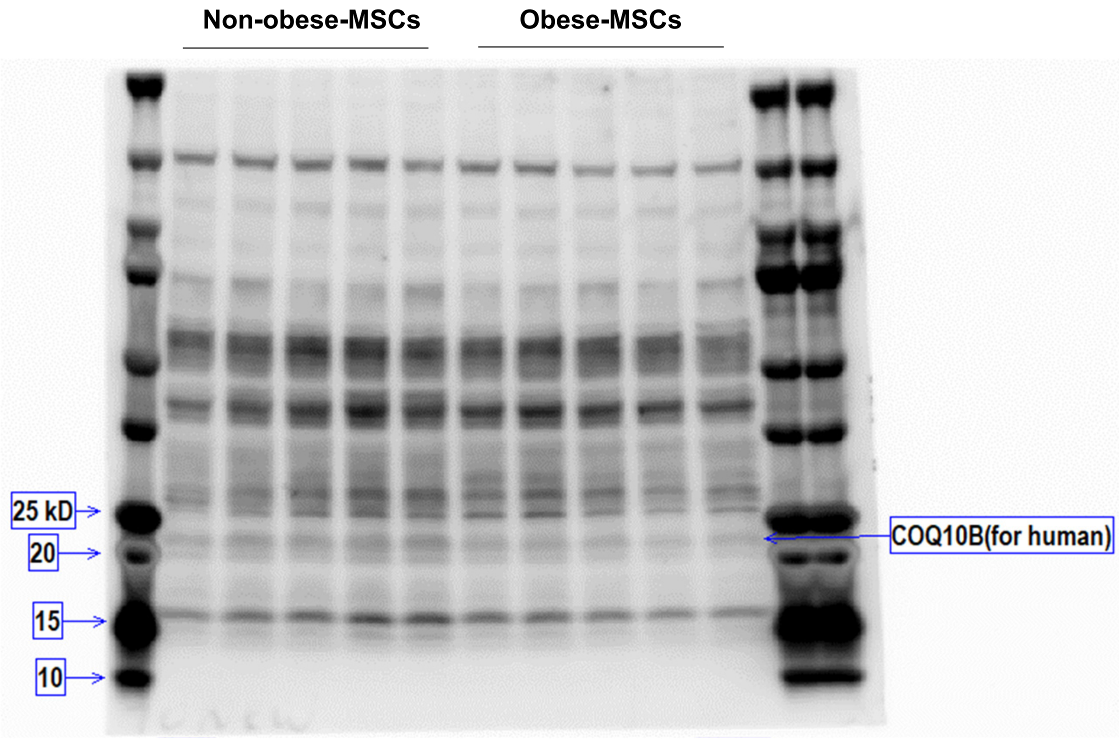

Fig. S6

LAMC1 (230kDa)

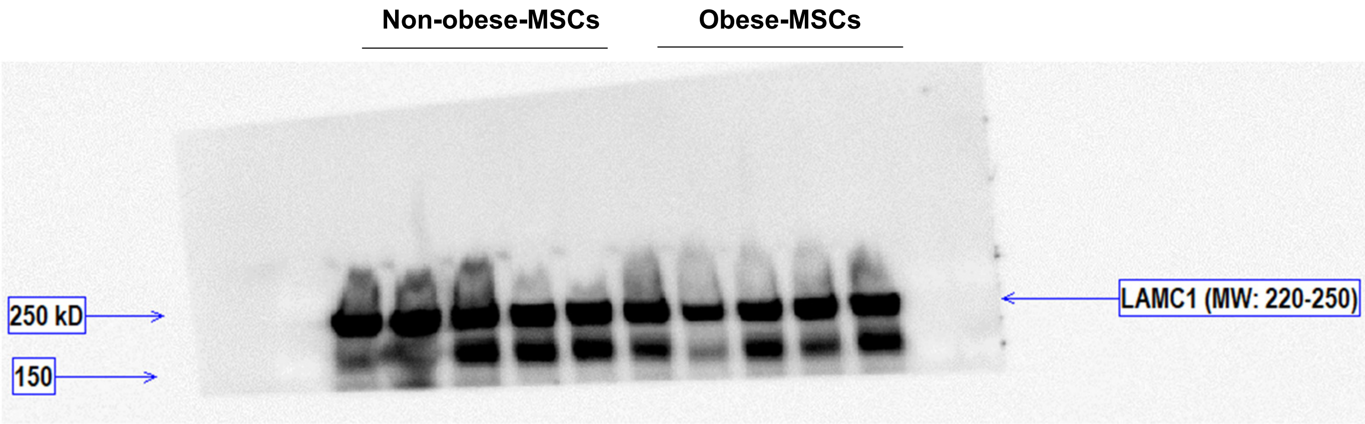

Fig. S7

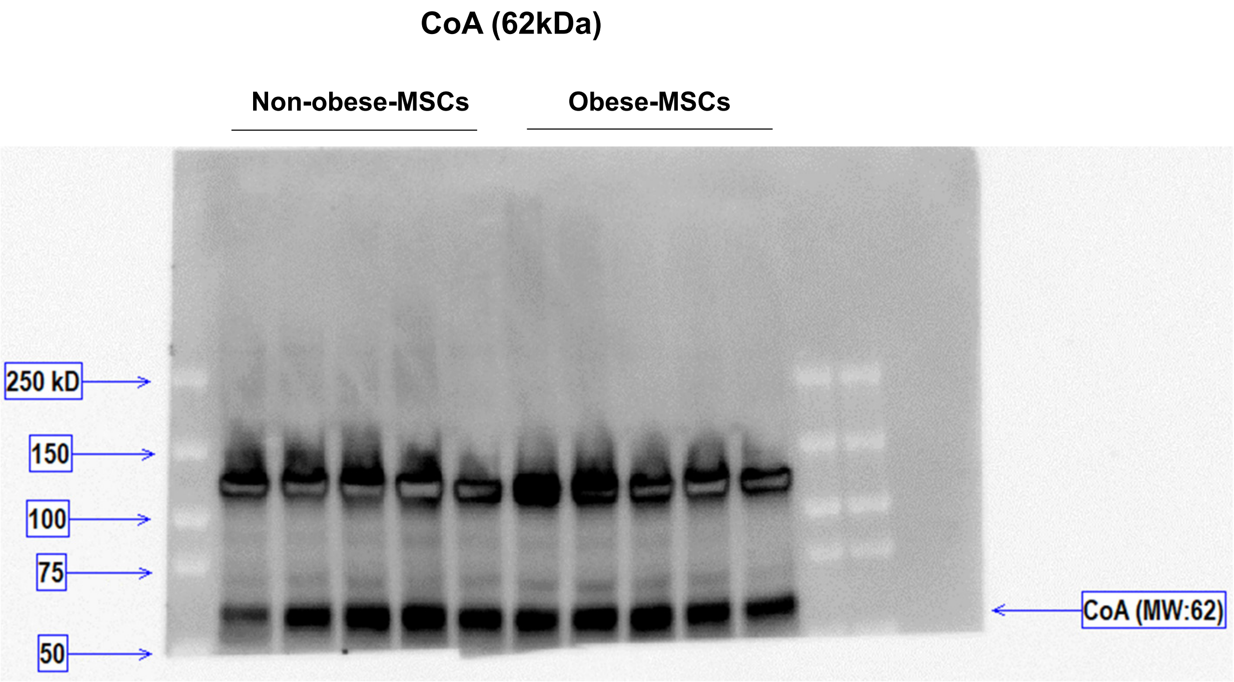

CoA (62kDa)
Non-obese-MSCs
Obese-MSCs
250 kD
150
100
75
50
CoA (MW:62)

Fig. S8

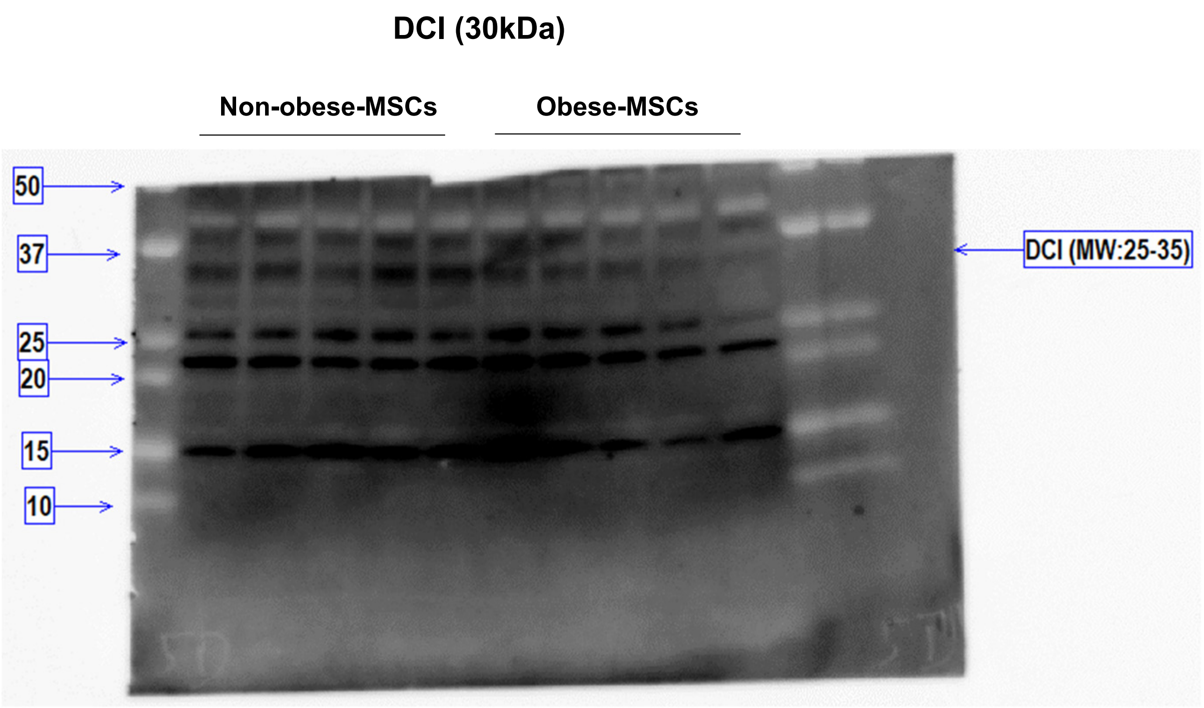
DCI (30kDa)
Non-obese-MSCs
Obese-MSCs
50
37
25
20
15
10
DCI (MW:25-35)

Fig. S9

MECR (35kDa)

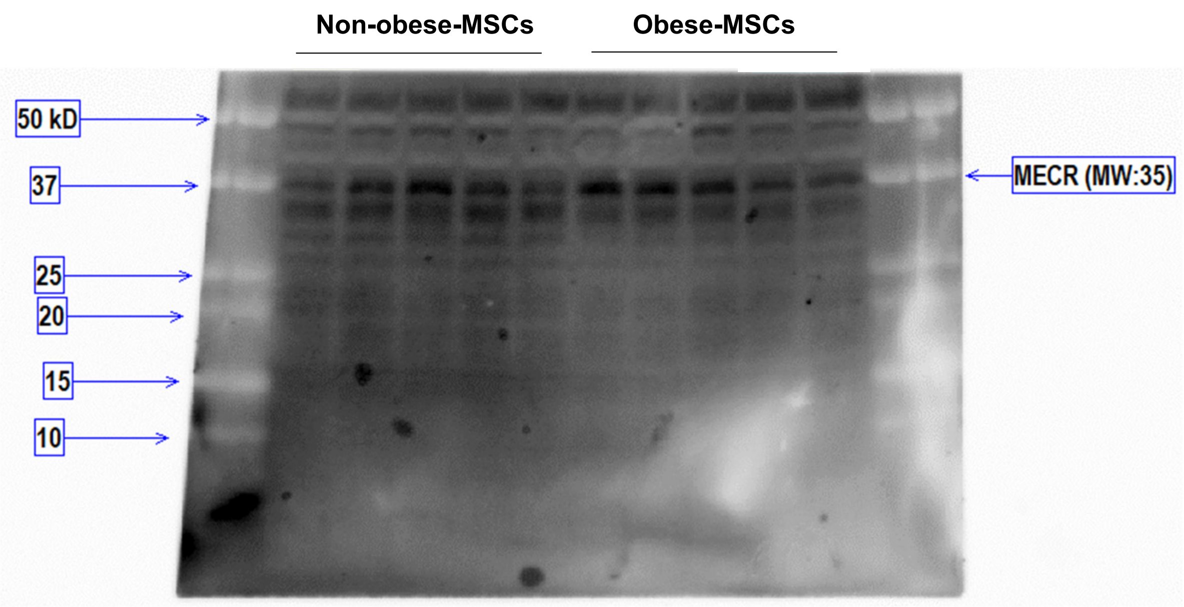

Fig. S10

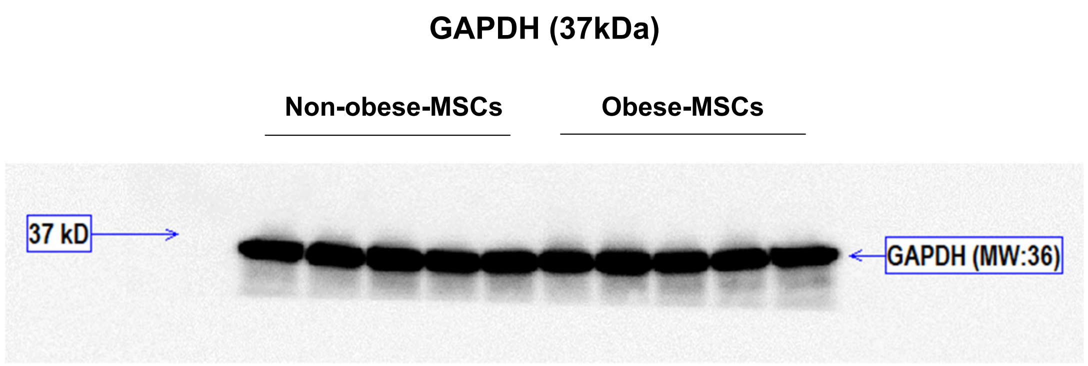

Fig. S11

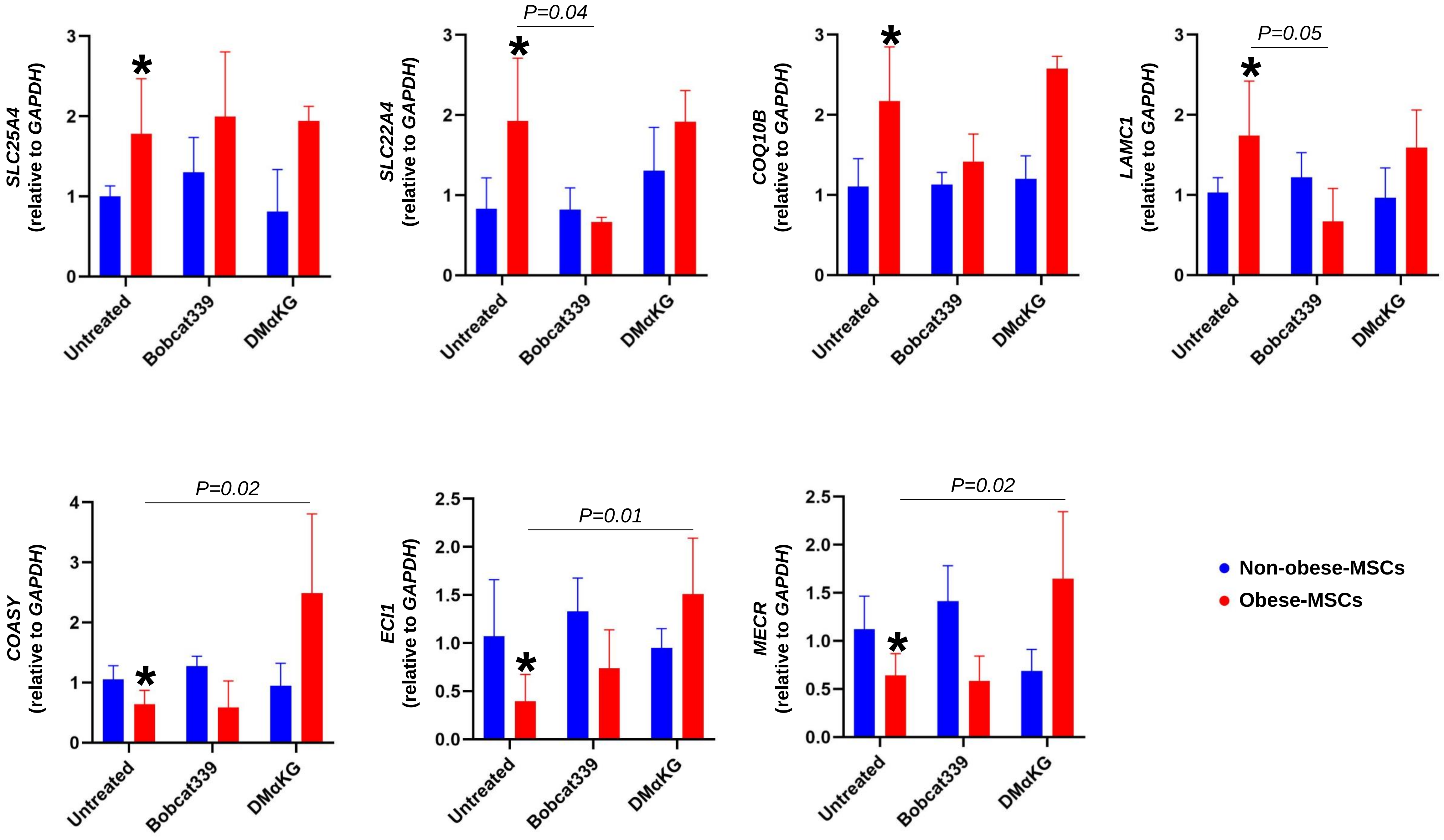
SLC25A4
(relative to GAPDH)
SLC22A4
(relative to GAPDH)
COQ10B
(relative to GAPDH)
LAMC1
(relative to GAPDH)
COASY
(relative to GAPDH)
ECI1
(relative to GAPDH)
MECR
(relative to GAPDH)
P=0.04
P=0.05
P=0.02
P=0.01
P=0.02
*
Untreated
Bobcat339
DMαKG
Non-obese-MSCs
Obese-MSCs

Table S1. Genes with significant higher 5hmC levels in obese- versus non-obese-MSCs. P values were adjusted by triglycerides levels, antihypertensives, and multivitamins.

Peank #	Gene Symbol	Chr	Start	End	Triglycerides (adj p value)	Antihypertensives (adj p value)	Multivitamins (adj p value)
1	FOXRED1	chr11	1.26E+08	1.26E+08	0.04995	0.00105	0.00117
2	TARS2	chr1	1.5E+08	1.5E+08	0.04974	0.00102	0.00113
3	ATXN2	chr12	1.11E+08	1.11E+08	0.04676	0.00092	0.00096
4	ELAC2	chr17	13003450	13004108	0.04650	0.00088	0.00095
5	CYP24A1	chr20	54173322	54173973	0.04646	0.00088	0.00092
6	CRY1	chr12	1.07E+08	1.07E+08	0.04607	0.00083	0.00086
7	GPX4	chr19	1105658	1106266	0.04574	0.00078	0.00084
8	ANGEL2	chr1	2.13E+08	2.13E+08	0.04563	0.00078	0.00084
9	AMT	chr3	49417818	49417973	0.04558	0.00076	0.00083
10	POLRMT	chr19	618997	619110	0.04072	0.00076	0.00082
11	MCU	chr10	72692619	72693066	0.03867	0.00074	0.00082
12	SARS2	chr19	38919762	38919999	0.03845	0.00074	0.00082
13	ACSM3	chr16	20610243	20610310	0.03823	0.00074	0.00080
14	RAB11FIP5	chr2	73156638	73156721	0.03818	0.00073	0.00079
15	PABPC5	chrX	91434595	91434894	0.03799	0.00072	0.00078
16	MRPS5	chr2	95106423	95106688	0.03726	0.00072	0.00078
17	ADCK5	chr8	1.44E+08	1.44E+08	0.03707	0.00069	0.00077
18	UQCRC1	chr3	48609497	48610037	0.03707	0.00065	0.00075
19	AIFM3	chr22	20974063	20974172	0.03427	0.00064	0.00073
20	MRPS36	chr5	69217760	69218115	0.03415	0.00064	0.00072
21	SLC25A11	chr17	4940381	4940993	0.03170	0.00063	0.00072
22	CPT1B	chr22	50572875	50573100	0.03154	0.00063	0.00071
23	COX6B2	chr19	55351699	55352326	0.02876	0.00062	0.00070
24	COQ10B	chr2	1.97E+08	1.97E+08	0.02854	0.00059	0.00070
25	SPATA20	chr17	50554251	50554450	0.02847	0.00058	0.00070
26	ACADSB	chr10	1.23E+08	1.23E+08	0.02810	0.00058	0.00068
27	AC114730.3	chr2	2.42E+08	2.42E+08	0.02805	0.00058	0.00068
28	BNIP3L	chr8	26422593	26422849	0.02683	0.00057	0.00066
29	CA5B	chrX	15738270	15738352	0.02677	0.00057	0.00066
30	ECHDC3	chr10	11755345	11755730	0.02645	0.00056	0.00066
31	SLC25A4	chr4	1.85E+08	1.85E+08	0.02605	0.00056	0.00062
32	RPS14	chr5	1.5E+08	1.5E+08	0.02576	0.00056	0.00061
33	VARS2	chr6	30923353	30924560	0.02571	0.00055	0.00060
34	TFAM	chr10	58385022	58385648	0.02512	0.00055	0.00060
35	CARS2	chr13	1.11E+08	1.11E+08	0.02402	0.00055	0.00060
36	OBSCN	chr1	2.28E+08	2.28E+08	0.02233	0.00054	0.00060
37	FASN	chr17	82085035	82085156	0.02177	0.00053	0.00059
38	LONP1	chr19	5690031	5692242	0.02173	0.00053	0.00059
39	SARS2	chr19	38916208	38916314	0.02159	0.00053	0.00059
40	QTRT1	chr19	10712663	10712867	0.02155	0.00052	0.00058
41	MRPL39	chr21	25607403	25607517	0.02039	0.00052	0.00058
42	CRAT	chr9	1.29E+08	1.29E+08	0.02016	0.00051	0.00057

43	OBSCN	chr1	2.28E+08	2.28E+08	0.02012	0.00051	0.00057
44	RHOT1	chr17	32141226	32142729	0.01946	0.00051	0.00056
45	FASN	chr17	82084513	82084716	0.01803	0.00051	0.00054
46	AGXT	chr2	2.41E+08	2.41E+08	0.01793	0.00050	0.00053
47	FPGS	chr9	1.28E+08	1.28E+08	0.01781	0.00050	0.00053
48	FDXR	chr17	74866446	74867005	0.01755	0.00050	0.00052
49	LIAS	chr4	39463525	39464238	0.01754	0.00049	0.00052
50	ECHDC2	chr1	52904646	52906611	0.01753	0.00049	0.00051
51	TMEM205	chr19	11342776	11343120	0.01677	0.00049	0.00051
52	NUCB2	chr11	17276984	17277095	0.01675	0.00049	0.00051
53	FASN	chr17	82088390	82088562	0.01599	0.00048	0.00050
54	NLRX1	chr11	1.19E+08	1.19E+08	0.01588	0.00048	0.00049
55	MTX1	chr1	1.55E+08	1.55E+08	0.01431	0.00048	0.00048
56	PACSIN2	chr22	42876137	42876333	0.01396	0.00048	0.00048
57	ATAD3B	chr1	1489204	1490671	0.01378	0.00047	0.00047
58	MECR	chr1	29200324	29200589	0.01361	0.00046	0.00047
59	MRPL51	chr12	6493058	6494154	0.01318	0.00046	0.00046
60	SARDH	chr9	1.34E+08	1.34E+08	0.01285	0.00046	0.00046
61	DHRSX	chrX	2243023	2243230	0.01259	0.00046	0.00046
62	DDX28	chr16	68023848	68024004	0.01253	0.00046	0.00045
63	GAPDH	chr12	6533927	6534661	0.01170	0.00045	0.00043
64	LDHD	chr16	75112602	75112713	0.01158	0.00045	0.00043
65	LAMC1	chr1	1.83E+08	1.83E+08	0.01157	0.00045	0.00042
66	FASN	chr17	82085158	82085402	0.01153	0.00044	0.00042
67	REXO2	chr11	1.14E+08	1.14E+08	0.01115	0.00044	0.00041
68	HAGH	chr16	1818515	1819223	0.01081	0.00042	0.00041
69	DCXR	chr17	82035858	82036063	0.01015	0.00041	0.00040
70	EARS2	chr16	23534888	23535449	0.00977	0.00041	0.00040
71	ABCF2	chr7	1.51E+08	1.51E+08	0.00970	0.00041	0.00040
72	SLC22A4	chr5	1.32E+08	1.32E+08	0.00947	0.00041	0.00040
73	MCEE	chr2	71109684	71110122	0.00937	0.00040	0.00039
74	LONP1	chr19	5694761	5694901	0.00922	0.00040	0.00039
75	MSRA	chr8	10411101	10411565	0.00911	0.00040	0.00039
76	REXO2	chr11	1.14E+08	1.14E+08	0.00799	0.00039	0.00038
77	FAHD2A	chr2	95410527	95411026	0.00756	0.00038	0.00038
78	FDXR	chr17	74865719	74865820	0.00735	0.00038	0.00038
79	AARS2	chr6	44305653	44305786	0.00729	0.00037	0.00037
80	TPI1	chr12	6869258	6869390	0.00725	0.00036	0.00036
81	ETFA	chr15	76230048	76230390	0.00634	0.00035	0.00036
82	WDR81	chr17	1730380	1730945	0.00632	0.00034	0.00034
83	PINK1	chr1	20644490	20644672	0.00587	0.00034	0.00034
84	ATPAF2	chr17	18018010	18018686	0.00576	0.00034	0.00034
85	CPS1	chr2	2.11E+08	2.11E+08	0.00566	0.00034	0.00033
86	SIRT3	chr11	236546	237144	0.00544	0.00033	0.00032
87	OXNAD1	chr3	16264595	16264972	0.00543	0.00033	0.00032
88	MTHFD1	chr14	64424804	64424931	0.00462	0.00032	0.00031

89	PTPMT1	chr11	47565274	47565986	0.00426	0.00030	0.00030
90	NIT1	chr1	1.61E+08	1.61E+08	0.00314	0.00027	0.00027
91	OPA1	chr3	1.94E+08	1.94E+08	0.00302	0.00027	0.00027
92	TCHP	chr12	1.1E+08	1.1E+08	0.00268	0.00026	0.00026
93	BCAT2	chr19	48796578	48796718	0.00196	0.00024	0.00023
94	PNPT1	chr2	55679682	55679795	0.00193	0.00023	0.00022
95	COX7A2L	chr2	42352887	42353343	0.00159	0.00022	0.00021
96	OBSCN	chr1	2.28E+08	2.28E+08	0.00154	0.00022	0.00021
97	SLC16A11	chr17	7042930	7043923	0.00122	0.00019	0.00019
98	NEU4	chr2	2.42E+08	2.42E+08	0.00092	0.00014	0.00015
99	AC005161.1	chr7	1.11E+08	1.11E+08	0.00043	0.00010	0.00009

Table S2. Genes with significant lower 5hmC levels in obese- versus non-obese-MSCs. P values were adjusted by triglycerides levels, antihypertensives, and multivitamins.

Peank #	Gene Symbol	Chr	Start	End	Triglycerides (adj p value)	Antihypertensives (adj p value)	Multivitamins (adj p value)
1	MRRF	chr9	122285169	122285946	0.00762	0.00039	0.00038
2	ACSL1	chr4	184770255	184770332	0.00660	0.00035	0.00036
3	DNM1L	chr12	32722427	32722772	0.00633	0.00035	0.00035
4	DHTKD1	chr10	12091513	12091684	0.00546	0.00034	0.00033
5	TTC19	chr17	16006474	16006568	0.00541	0.00033	0.00032
6	APOPT1	chr14	103607204	103607523	0.00493	0.00033	0.00031
7	MRPL14	chr6	44113454	44114209	0.00443	0.00032	0.00030
8	HIBADH	chr7	27531192	27531348	0.00440	0.00031	0.00030
9	NCOA4	chr10	46005088	46006597	0.00427	0.00031	0.00030
10	RHOT2	chr16	667432	667833	0.00425	0.00030	0.00029
11	IMMP2L	chr7	110724159	110724591	0.00423	0.00030	0.00029
12	GPAM	chr10	112214319	112214396	0.00393	0.00029	0.00029
13	MTCP1	chrX	155065486	155065537	0.00367	0.00029	0.00029
14	MRPS22	chr3	139348160	139348507	0.00365	0.00029	0.00029
15	UCP1	chr4	140559434	140560010	0.00350	0.00029	0.00028
16	GFM1	chr3	158684524	158685314	0.00345	0.00029	0.00028
17	EEFSEC	chr3	128336904	128337092	0.00336	0.00028	0.00028
18	OPA1	chr3	193691394	193692132	0.00320	0.00028	0.00028
19	HSD17B4	chr5	119535770	119536550	0.00316	0.00028	0.00028
20	SLIRP	chr14	77717599	77718530	0.00305	0.00027	0.00027
21	COASY	chr17	42561467	42561680	0.00288	0.00027	0.00027
22	PDSS2	chr6	107458990	107459564	0.00285	0.00027	0.00027
23	FASN	chr17	82093219	82093419	0.00277	0.00026	0.00026
24	DAAM2	chr6	39899578	39900071	0.00252	0.00026	0.00026
25	SUOX	chr12	55999333	55999590	0.00248	0.00025	0.00025
26	COA3	chr17	42796621	42796936	0.00225	0.00024	0.00025
27	IKZF5	chr10	122997119	122997513	0.00224	0.00024	0.00025
28	STARD7	chr2	96195203	96195549	0.00216	0.00024	0.00024
29	ABCA13	chr7	48278094	48279920	0.00215	0.00024	0.00024
30	DLAT	chr11	112028792	112028945	0.00213	0.00024	0.00024
31	ECI1	chr16	2239395	2240145	0.00211	0.00024	0.00023
32	SLC25A3	chr12	98593591	98595848	0.00209	0.00024	0.00023
33	GLDC	chr9	6532464	6533160	0.00194	0.00024	0.00022
34	PC	chr11	66848233	66849147	0.00191	0.00023	0.00022
35	PI4KA	chr22	20747484	20747702	0.00184	0.00023	0.00022
36	GATM	chr15	45361124	45362221	0.00179	0.00023	0.00021
37	GLYCTK	chr3	52288580	52289276	0.00179	0.00023	0.00021
38	ABCB9	chr12	122959635	122960322	0.00176	0.00023	0.00021
39	MMADHC	chr2	149569634	149570168	0.00173	0.00023	0.00021
40	ACSM5	chr16	20440266	20441586	0.00172	0.00023	0.00021
41	ACADL	chr2	210203331	210203444	0.00164	0.00022	0.00021
42	SLC22A4	chr5	132294443	132295009	0.00147	0.00022	0.00021

43	OXNAD1	chr3	16294856	16294997	0.00141	0.00022	0.00020
44	LRPPRC	chr2	43901320	43903931	0.00139	0.00021	0.00020
45	GFM1	chr3	158645822	158646297	0.00138	0.00020	0.00020
46	LAMC1	chr1	183125188	183125550	0.00137	0.00020	0.00019
47	MRPS5	chr2	95087207	95087581	0.00134	0.00020	0.00019
48	LYRM1	chr16	20920122	20920674	0.00132	0.00020	0.00019
49	BDH1	chr3	197538306	197538484	0.00127	0.00020	0.00019
50	AC007528.1	chr12	16409255	16409331	0.00125	0.00019	0.00019
51	NT5DC3	chr12	103785571	103785747	0.00115	0.00018	0.00018
52	CARS2	chr13	110642315	110645633	0.00111	0.00018	0.00018
53	KIAA0100	chr17	28640397	28640683	0.00109	0.00017	0.00018
54	NMNAT3	chr3	139560180	139561392	0.00108	0.00017	0.00018
55	METAP1D	chr2	172070663	172071070	0.00108	0.00017	0.00017
56	MRPS27	chr5	72227050	72229553	0.00107	0.00016	0.00017
57	DHCR24	chr1	54871350	54871613	0.00107	0.00016	0.00017
58	MAOB	chrX	43766611	43767618	0.00107	0.00016	0.00017
59	PACSIN2	chr22	42912021	42912157	0.00102	0.00015	0.00017
60	LYRM1	chr16	20924000	20925006	0.00098	0.00015	0.00017
61	TRAP1	chr16	3676431	3676946	0.00096	0.00015	0.00016
62	CROT	chr7	87398524	87399795	0.00094	0.00015	0.00016
63	TXNRD1	chr12	104262314	104262575	0.00094	0.00015	0.00016
64	PREPL	chr2	44359497	44360676	0.00091	0.00014	0.00015
65	NDUFAF4	chr6	96889313	96891391	0.00085	0.00014	0.00015
66	GATC	chr12	120459907	120463749	0.00081	0.00014	0.00015
67	ABCB9	chr12	122948624	122949312	0.00080	0.00014	0.00014
68	SFXN3	chr10	101031073	101031157	0.00080	0.00014	0.00014
69	SLC25A12	chr2	171784370	171785475	0.00075	0.00014	0.00013
70	EMC2	chr8	108473851	108475963	0.00075	0.00014	0.00013
71	TIMMDC1	chr3	119523606	119525749	0.00074	0.00014	0.00013
72	PCCA	chr13	100530098	100532601	0.00072	0.00013	0.00013
73	GLOD4	chr17	783083	783517	0.00072	0.00013	0.00012
74	FAM162A	chr3	122401376	122401535	0.00071	0.00013	0.00012
75	SPG7	chr16	89548003	89548378	0.00070	0.00013	0.00012
76	NLN	chr5	65785775	65786060	0.00069	0.00012	0.00012
77	RDH13	chr19	55056653	55056808	0.00069	0.00012	0.00012
78	MCU	chr10	72859177	72859347	0.00069	0.00012	0.00012
79	NT5DC3	chr12	103788838	103789057	0.00069	0.00011	0.00012
80	MGST3	chr1	165648450	165648670	0.00066	0.00011	0.00011
81	ACSL1	chr4	184808292	184808601	0.00063	0.00011	0.00011
82	MECR	chr1	29223140	29223549	0.00061	0.00011	0.00011
83	GDAP1	chr8	74363985	74366876	0.00061	0.00011	0.00011
84	QRSL1	chr6	106652467	106653823	0.00058	0.00011	0.00011
85	TRAP1	chr16	3656964	3657341	0.00057	0.00011	0.00010
86	ECH1	chr19	38830904	38831166	0.00057	0.00011	0.00010
87	CYP11A1	chr15	74337759	74338103	0.00055	0.00011	0.00010
88	DDAH1	chr1	85398457	85399963	0.00051	0.00011	0.00010

89	RP11-60I3.4	chr9	99928778	99929169	0.00050	0.00010	0.00010
90	ABHD10	chr3	111991377	111993363	0.00046	0.00010	0.00009
91	ACADM	chr1	75762692	75763679	0.00043	0.00010	0.00009
92	C15orf40	chr15	82989881	82991057	0.00042	0.00010	0.00009
93	DNAJA3	chr16	4440257	4441575	0.00040	0.00010	0.00009
94	SSBP1	chr7	141737383	141737775	0.00034	0.00010	0.00009
95	MAOB	chrX	43769307	43769418	0.00033	0.00010	0.00009
96	LIPT1	chr2	99154291	99154636	0.00032	0.00010	0.00008
97	MRRF	chr9	122330867	122331343	0.00031	0.00009	0.00008
98	PNPLA8	chr7	108554543	108554859	0.00031	0.00009	0.00008
99	SLC25A20	chr3	48859092	48859201	0.00031	0.00008	0.00008
100	MRPS5	chr2	95108175	95108408	0.00030	0.00008	0.00008
101	NDUFAF7	chr2	37241578	37242693	0.00028	0.00008	0.00008
102	MUTYH	chr1	45334100	45334511	0.00027	0.00007	0.00007
103	EPHX2	chr8	27516320	27516398	0.00027	0.00007	0.00007
104	SAMM50	chr22	43964452	43964553	0.00026	0.00007	0.00007
105	NDUFA2	chr5	140644573	140645725	0.00026	0.00007	0.00007
106	ABCA13	chr7	48352181	48352487	0.00026	0.00007	0.00007
107	NNT	chr5	43612908	43613650	0.00026	0.00006	0.00006
108	AK2	chr1	33007984	33010833	0.00025	0.00006	0.00006
109	LYRM4	chr6	5236284	5236461	0.00023	0.00006	0.00006
110	PDHB	chr3	58430657	58433857	0.00023	0.00006	0.00006
111	DLAT	chr11	112026198	112026299	0.00022	0.00006	0.00006
112	C15orf40	chr15	82988441	82988828	0.00021	0.00006	0.00006
113	PPOX	chr1	161167100	161167486	0.00020	0.00006	0.00005
114	TYSND1	chr10	70143842	70144835	0.00019	0.00005	0.00005
115	PCK2	chr14	24099558	24100562	0.00015	0.00005	0.00005
116	TEFM	chr17	30898908	30900562	0.00015	0.00005	0.00005
117	MRPL11	chr11	66435075	66436112	0.00014	0.00005	0.00005
118	CPOX	chr3	98579442	98580770	0.00014	0.00005	0.00005
119	GSTK1	chr7	143269292	143270854	0.00013	0.00005	0.00005
120	AIFM2	chr10	70121092	70121211	0.00013	0.00005	0.00004
121	ECI1	chr16	2242415	2243439	0.00013	0.00004	0.00004
122	NFS1	chr20	35673758	35674431	0.00011	0.00004	0.00004
123	RAB24	chr5	177301198	177301807	0.00011	0.00004	0.00004
124	RPS15A	chr16	18781295	18786795	0.00011	0.00003	0.00004
125	ACOT13	chr6	24700907	24706262	0.00009	0.00003	0.00004
126	MRPL39	chr21	25601368	25601467	0.00009	0.00003	0.00003
127	OBSCN	chr1	228302389	228302998	0.00008	0.00002	0.00003
128	ALDH7A1	chr5	126541841	126545019	0.00007	0.00002	0.00003
129	METTL15	chr11	28330396	28333507	0.00007	0.00002	0.00002
130	KIF1B	chr1	10376545	10381603	0.00006	0.00002	0.00002
131	ALDH7A1	chr5	126594108	126594439	0.00006	0.00002	0.00002
132	COX20	chr1	244839747	244845344	0.00006	0.00002	0.00002
133	MTO1	chr6	73480997	73481116	0.00006	0.00002	0.00002
134	SUGCT	chr7	40860316	40860763	0.00005	0.00002	0.00002

135	SND1	chr7	127857852	127858488	0.00004	0.00002	0.00002
136	MTCH1	chr6	36969466	36970114	0.00004	0.00001	0.00002
137	C15orf61	chr15	67521068	67522242	0.00003	0.00001	0.00001
138	NDUFAF5	chr20	13817118	13821582	0.00003	0.00001	0.00001
139	RMDN3	chr15	40738501	40739680	0.00003	0.00001	0.00001
140	C8orf82	chr8	144529179	144530616	0.00003	0.00001	0.00001
141	COX15	chr10	99713515	99714718	0.00002	0.00001	0.00001
142	FABP1	chr2	88126485	88126746	0.00002	0.00001	0.00001
143	EARS2	chr16	23522014	23524454	0.00001	0.00001	0.00001
144	ACAD9	chr3	128912507	128913114	0.00001	0.00000	0.00000
145	NDUFAF5	chr20	13803150	13803421	0.00001	0.00000	0.00000
146	SLC25A16	chr10	68499532	68499968	0.00001	0.00000	0.00000
147	IFI27	chr14	94116442	94116698	0.00000	0.00000	0.00000
148	RPS15A	chr16	18788063	18788159	0.00000	0.00000	0.00000
149	SLC25A28	chr10	99610522	99611366	0.00000	0.00000	0.00000
150	MPC1	chr6	166365974	166366106	0.00000	0.00000	0.00000
